# Study on changes in the physiological growth characteristics and yield-increasing effects of winter wheat under water-fertilizer-pesticide synergistic regulation

**DOI:** 10.3389/fpls.2026.1865682

**Published:** 2026-07-01

**Authors:** Jianqin Ma, Yiyi Zhang, Bifeng Cui, Xiuping Hao, Yu Ding, Chenyuan Wei

**Affiliations:** College of Water Resources, North China University of Water Resources and Electric Power, Zhengzhou, China

**Keywords:** growth characteristics, modified logistic model, pesticide, photosynthesis, winter wheat, yield

## Abstract

**Introduction:**

Against the backdrop of global climate change and resource constraints, the extensive management of water, fertilizer, and pesticide resources has exacerbated non-point source pollution in farmland. The patterns of crop pests and diseases are becoming increasingly complex, and relying solely on chemical control methods is insufficient to meet the demands of sustainable pest management. This has led to constraints on crop growth and a significant decline in yields. To promote the transition of crop protection practices toward a green and sustainable model, this study conducted field trials on water-fertilizer-pesticide synergistic regulation (WFPSR) in the North China Plain from 2023-2025.

**Methods:**

Four reduced-rate application levels were set (R1: 14%, R2: 33%, R3: 53%, R4: 73%), two real-time irrigation levels (W1: 80% θ_fc_-60% θ_fc_, W2: 90% θ_fc_-70% θ_fc_), two fertilizer application types (conventional compound fertilizer, water-soluble fertilizer + microbial fertilizer), and a control treatment (CK) using local standard application rates for water, fertilizer, and pesticides, resulting in a total of 9 WFPSR combinations. This study analyzes the physiological growth characteristics of winter wheat under water-fertilizer-pesticide synergy using fitting results based on an improved logistic model, and investigates the relationship between crop physiological growth characteristics and yield.

**Results and discussion:**

Appropriate WFPSR significantly promoted the physiological growth of winter wheat, with the W1R2 treatment yielding the best results. Compared to CK, the SPAD, Pn, and LAI values of the W1R2 treatment increased by 17.3%, 11.3%, and 7.84%, respectively. The Logistic model fit well, with R² values all exceeding 0.95. PCA-based dimensionality reduction and yield fitting models indicated that the W1R2 treatment increased yields by improving crop physiological functions, thereby increasing thousand-kernel weight and the number of grains per spike. With yield, water and fertilizer savings, and pesticide savings increasing by 8.2%, 40.2%, and 33%, respectively, compared to CK. In summary, the WFPSR model based on the W1R2 combination provides a theoretical foundation for the integrated management of green yield increases in winter wheat on the North China Plain.

## 1 Introduction

As one of the world’s three major food crops, wheat plays a vital role in food production (Acevedo et al., 2018). Agricultural production, led by wheat, accounts for as much as 70% of global water consumption (Loaiciga and Doh, 2024; Karandish et al., 2025). Of the more than 3.7 million metric tons of pesticides used worldwide each year, approximately 60% remain in the soil due to poor management (Faostat, 2024), causing environmental pollution (Sun et al., 2018) while also affecting normal crop growth and yields (Tudi et al., 2021). As one of China’s most important grain-producing regions, the North China Plain suffers from low water use efficiency due to traditional, extensive irrigation practices (Si et al., 2020). Furthermore, long-term straw return to the fields and climatic factors have led to frequent outbreaks of soil-borne diseases such as stem-base rot (Deng et al., 2020). To control pests and diseases, traditional intensive agricultural practices often involve the overuse of chemical pesticides; agricultural pollution characterized by chemical fertilizer and pesticide residues has further exacerbated water eutrophication and soil degradation on the North China Plain, creating a dual burden of water scarcity and environmental pollution (Steenhuis and Yang, 2021; Xia et al., 2023). With multiple challenges intertwined, traditional, single-factor, extensive management approaches are no longer sufficient to meet the demands of sustainable production, making it imperative for China’s agricultural production system to promote the adoption of efficient water-saving technologies and strengthen green pest and disease control measures.

As an advanced precision agriculture technology, real-time water-fertilizer coordination technology combines Internet of Things (IoT) technology with drip irrigation and integrated water-fertilizer systems to enable precise control of irrigation and fertilization (Bwambale et al., 2022). It effectively reduces crop evapotranspiration and improves water and fertilizer use efficiency, offering a viable technical solution to challenges such as water scarcity, frequent disease outbreaks, and the overuse of pesticides. Currently, scholars both domestically and internationally are conducting research on reducing pesticide application rates in agricultural fields. Zanin et al. (2022) reported that the use of site-specific spraying technology reduced the total amount of pesticides applied without significantly affecting the yields of soybean and corn crops. Kamran et al. (2018) noted that reducing the application of tebuconazole by 30% in wheat led to significant improvements in various stem morphological traits, such as stem diameter and stem filling. Santos et al. (2022) confirmed that the combined application of Bacillus subtilis preparations with chemical fungicides not only significantly reduced the severity of crop diseases but also achieved control efficacy comparable to conventional chemical-only treatments, resulting in a 25% increase in crop yield. Wei et al. (2024) confirmed that the combined use of biocontrol agents and chemical fungicides exhibits a significant synergistic effect. This combined strategy not only resulted in disease control efficacy significantly superior to that of conventional chemical pesticides applied at standard doses but also successfully reduced the application rate of chemical pesticides by 50%. On the other hand, a study by Tudi et al. (2021) found that excessive irrigation of farmland accelerates the leaching of pesticides into deeper soil layers and groundwater, reducing their efficacy while also exacerbating non-point source pollution. Regarding growth models, Chang et al. (2023) established a dynamic normalized logistic model describing the LAI of maize by substituting accumulated temperature for growing days. Jiang et al. (2020) used a modified logistic model combining temperature and water response functions to accurately simulate and analyze the growth dynamics of winter wheat plant height under different soil water stress conditions. Yan et al. (2022) used a logistic model to analyze the dynamic characteristics of aboveground dry matter accumulation in winter wheat under water-fertility coupling conditions.

In summary, the effects of different pesticide reduction gradients, the application of microbial biofertilizers, and irrigation gradients on wheat physiological growth indicators and yield are highly complex. This underscores the need to identify the optimal water-fertilizer-pesticide synergistic regulation (WFPSR), with the aim of ensuring normal wheat growth while maximizing yield, water and fertilizer use efficiency, and pesticide use efficiency. Therefore, we hypothesize that different levels of real-time irrigation and fertilizer-pesticide treatments have a significant impact on the physiological growth characteristics and yield of winter wheat. Integrated management combining moderate real-time irrigation with a specific threshold for pesticide reduction and the application of microbial biofertilizers can significantly optimize crop growth dynamics, enhance plant stress resistance, and ultimately achieve the dual goals of sustainable yield increases.

Existing research has largely focused on the regulation of individual factors, using growth models to study crop growth and development dynamics under conditions where only a single factor—such as water or nutrients—is controlled, or examining the response patterns of crop growth traits to pesticide reduction in isolation. The mechanisms underlying the synergistic interaction among multiple factors—including water, fertilizer, and pesticides—remain poorly understood, and there is a particular lack of quantitative studies on crop physiological responses to the combination of precision irrigation and fertilizer-pesticide treatments based on real-time feedback. To address the aforementioned research gaps, this study conducted WFPSR trials over two growing seasons (2023–2024 and 2024-2025), with the following objectives: (1) To clarify the effects of WFPSR on the physiological growth indicators and yield of winter wheat; (2) To investigate the dynamic changes in physiological growth characteristics under different treatments using an improved logistic growth model, and to analyze the relationships among yield, yield components, and various physiological growth indicators; (3) To propose an optimal WFPSR.

## 2 Materials and methods

### 2.1 Overview of the test area

**Figure 1:**
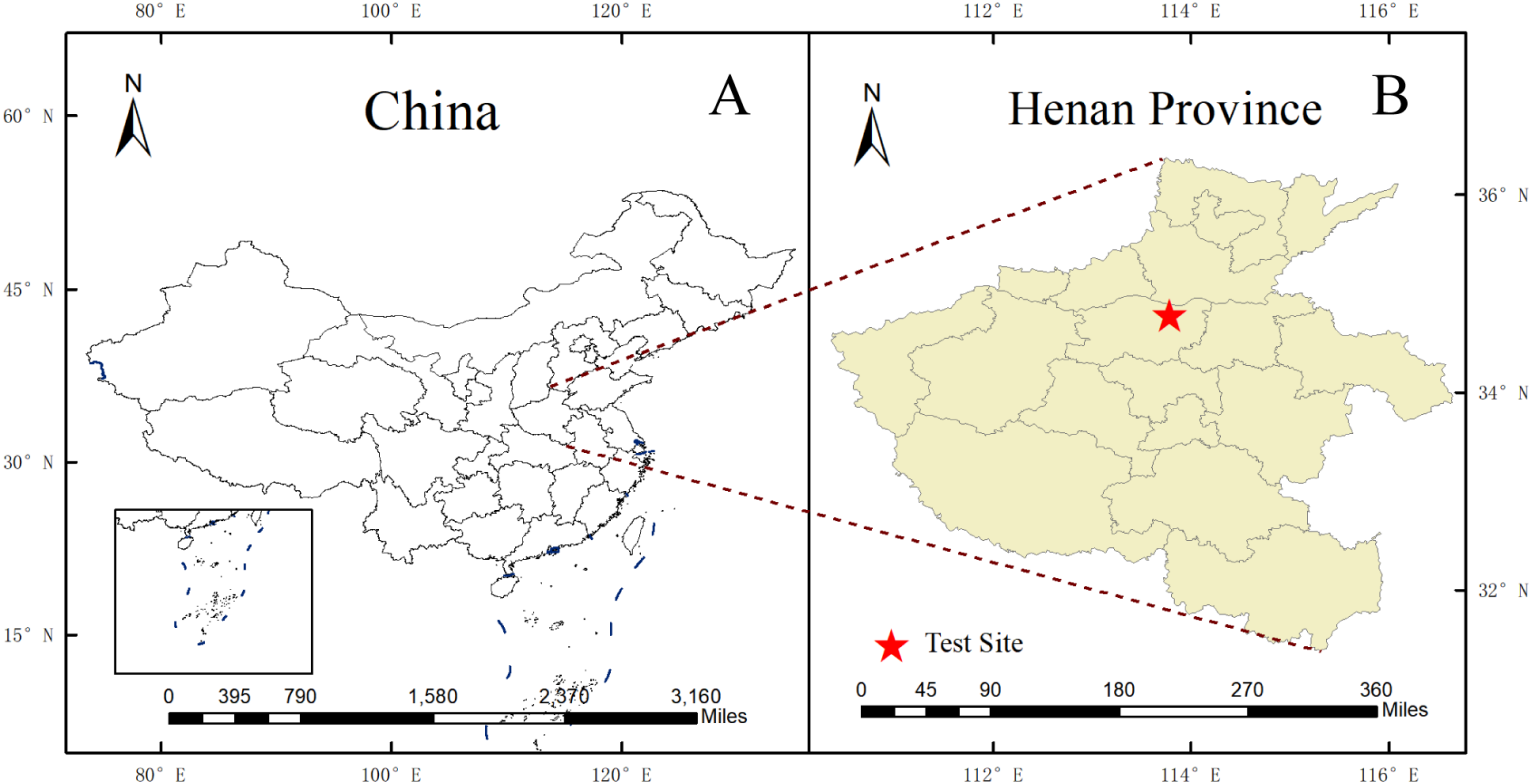
Location map of the study area. **(A)** is China; **(B)** is Henan Province.

**Figure 2:**
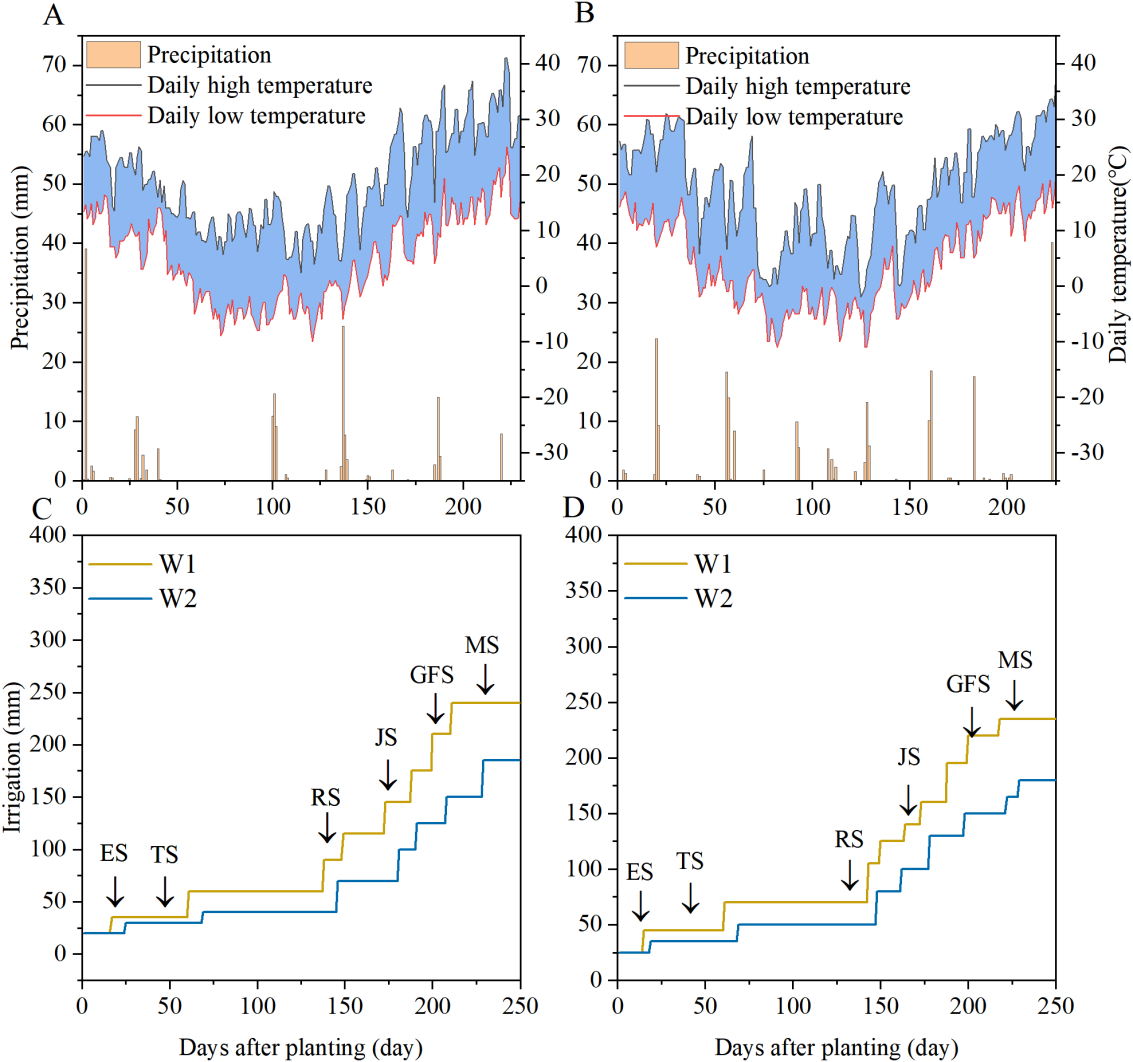
Changes in meteorological conditions and irrigation timing during the 2023–2025 winter wheat growing seasons. **(A, B)** show changes in daily temperature and precipitation during the 2023–2025 winter wheat growing seasons, and **(C, D)** show irrigation volumes for different real-time irrigation treatments during the 2023–2025 winter wheat growing seasons. ES, TS, RS, JS, GFS, and MS represent the emergence, tillering, regreening, jointing, grain filling, and maturity stages of wheat, respectively.

The experiment was conducted at the Agricultural Efficient Water Use Irrigation Experimental Station on the Longzi Lake Campus of North China University of Water Resources and Electric Power in Zhengzhou, Henan Province (latitude 34.8°N, longitude 113.9°E) (**Figure 1**). Rainfall in the experimental area is primarily concentrated between June and August. The annual average precipitation is 632.4 mm, with a maximum annual precipitation of 1,339 mm and a minimum annual precipitation of 380.6 mm. The average temperature is 14.7 °C, with an extreme minimum temperature of -16.3 °C and an extreme maximum temperature of 41.5 °C. The distribution of rainfall and temperature in the field after wheat sowing is shown in **Figure 2**. The soil texture of the experimental plot’s topsoil is loam, with a field moisture content of 31.65%, a bulk density of 1.48 g/cm^3^, and a soil electrical conductivity of 0.322 dS/m. The total nitrogen content is 460 mg/kg, the available phosphorus content is 17.22 mg/kg, the available potassium content is 79 mg/kg, the organic matter content is 17.3 g/kg, and the soil pH is 8.2.

### 2.2 Experimental design

A total of 9 water-fertilizer-pesticide application treatments were established in the 2023-2025 (2023–2024 and 2024–2025 growing seasons) field trials (**Table 1**), with two real-time irrigation levels selected (W1: 80%θ*_fc_*-60%θ*_fc_*, W2: 90%θ*_fc_*-70%θ*_fc_*). For soil-borne stem base rot disease in winter wheat, treatment R1 followed the “Guidelines for Rational Use of Pesticides” (State Administration of the People's Republic of China, 2018) and high-standard farmland specifications for recommended pesticide types and application rates. The control treatment (CK) used pesticides and application rates based on local farmers’ experience. Based on CK, the pesticide application reduction gradients were designed as follows: R2: 33% reduction from local farmer’s empirical application rate; R3: 53% reduction; R4: 73% reduction. The types of fertilizers include water-soluble fertilizers, microbial fertilizers, and conventional fertilizers. A total of 9 treatments were established under the WFPSR, each with 3 replicates, for a total of 27 plots. Each plot measured 15 m^2^ (5 m × 3 m) and was arranged in a randomized block design.

**Table 1:** Water-fertilizer-pesticide experiment design, 2023–2025.

No	Irrigation level (W)		Fertilizer-pesticide treatment (R)			Treatment
	Upper limit of irrigation	Lower limit of irrigation	Types of pesticide application	Pesticide application rate	Types of fertilizer	
1	80%*θ_fc_*(W1)	60%*θ_fc_*	40% Prothioconazole·Tebuconazole	Standard application rate (525 ml·ha^-1^)	Water-soluble fertilizer	W1R1
2				Local farmers’ experience application rate reduced by 33%(405ml·ha^-1^)	Water-soluble fertilizer + Microbial fertilizer	W1R2
3				Local farmers’ experience application rate reduced by 53%(285ml·ha^-1^)	Water-soluble fertilizer + Microbial fertilizer	W1R3
4				Local farmers’ experience application rate reduced by 73%(165ml·ha^-1^)	Water-soluble fertilizer + Microbial fertilizer	W1R4
5	90%*θ_fc_*(W2)	70%*θ_fc_*	40% Prothioconazole·Tebuconazole	Standard application rate(525ml·ha^-1^)	Water-soluble fertilizer	W2R1
6				Local farmers’ experience application rate reduced by 33%(405ml·ha^-1^)	Water-soluble fertilizer + Microbial fertilizer	W2R2
7				Local farmers’ experience application rate reduced by 53%(285ml·ha^-1^)	Water-soluble fertilizer + Microbial fertilizer	W2R3
8				Local farmers’ experience application rate reduced by 73%(165ml·ha^-1^)	Water-soluble fertilizer + Microbial fertilizer	W2R4
9	Local Experience Irrigation Amount		27% Propiconazole·Thiabendazole	Local farmers’ experience application rate(900ml·ha^-1^)	Conventional Fertilizer	CK

Based on calculations performed using test samples, θ*_fc_* = 31.65.

Each plot is supplied with water via a surface drip irrigation system, which, in conjunction with field monitoring devices (WSH-TDR310S) that measure soil moisture content (θ*_fc_*) in real time, enables automatic irrigation. Sensors for the monitoring devices were installed at depths of 20 cm, 40 cm, and 60 cm within the crop root zone. Monitoring was conducted every 30 minutes, and the weighted average was calculated as a control parameter to guide the smart device in determining irrigation settings (Ma et al., 2015). Irrigation was initiated when the measured value fell below the preset lower limit and terminated when it reached or exceeded the preset upper limit. Irrigation volume was calculated based on water meter readings before and after irrigation, with the results shown in **Figure 2**. The CK group field irrigation employed local empirical irrigation rates and methods.

The specific application strategy was formulated based on disease control practices for wheat cultivation in the North China Plain, primarily targeting the soil-borne disease stem base rot in winter wheat. The selected fungicide is 40% propiconazole·tebuconazole (525 ml·ha^-1^). Research indicates that local farmers predominantly apply 27% propiconazole·thiabendazole (900 ml·ha^-1^). Application timing and method: foliar spraying during wheat jointing stage. Given that the initial soil pH at the experimental site was relatively high, indicating alkaline conditions, well-decomposed organic fertilizer (12,000 kg/ha) and gypsum (600 kg/ha) were applied uniformly to all experimental plots as a basal dressing 14 days prior to sowing to mitigate soil alkalinity and optimize the rhizosphere microenvironment (Bello et al., 2021), which was then incorporated into the 0–20 cm tillage layer through rotary tilling and thoroughly mixed prior to sowing. For CK, conventional compound fertilizer was broadcast-applied combined with water-soluble fertilizer via drip irrigation. Other pesticide reduction treatments (R1, R2, R3, R4) applied conventional fertilizer were applied via drip irrigation. In treatments R2, R3, and R4, microbial inoculant solutions (primary strains: *Bacillus subtilis*, *Bacillus amyloliquefaciens*, etc.) were selected for drip irrigation application throughout the entire growth cycle. The application rate was 7500 ml·ha^-1^, administered at three key growth stages: emergence, jointing, and heading. This approach aimed to reduce crop diseases, enhance plant stress resistance, and achieve pesticide reduction objectives. The winter wheat variety used in the experiment was “Fanmai No. 8.” This variety is widely cultivated on the North China Plain and offers high yield potential as well as relatively good cold and drought tolerance. In this experiment, the variety was sown in mid- to late October each year and harvested in early June of the following year.

### 2.3 Test items and methods

#### 2.3.1 Soil moisture content

The WSH-TDR310S sensor is used for real-time measurement of soil moisture content. This device operates on the principle of time-domain reflectometry and features a 10-centimeter probe. Its measurement range is 0-100% volumetric moisture content (accuracy: ± 2%, resolution: 0.1%).

#### 2.3.2 Meteorological indicators

The meteorological data is collected using the JianDa RenKe RS-QXZN-M3 compact automatic weather station, which can simultaneously monitor and transmit real-time data on 15 meteorological parameters, including air temperature, humidity, wind speed, wind direction, atmospheric pressure, precipitation, and total radiation.

#### 2.3.3 Growth indicators

Three samples were collected from wheat plants in each treatment plot. Plant height was measured using a tape measure (measured to the natural height of the plant before the jointing stage, and subsequently measured to the tip of the spike; units: cm). The samples were blanched and dried to constant weight, and the accumulated dry matter of the wheat was determined using the traditional weighing method.

Field sampling is conducted during key physiological growth stages throughout the crop growth cycle. The sampling dates for 2023–2024 are: November 5 (emergence stage), November 18 (tillering stage), March 5 (regreening stage), April 10 (jointing stage), May 4 (grain-filling stage), and June 1 (maturity stage). The sampling dates for 2024–2025 are: November 3, November 15, March 9, April 6, May 3, and May 29.

#### 2.3.4 Physiological indicators

After wheat emergence, photosynthetic measurements were conducted at key physiological growth stages. From each plot and replicate, five plants of the same leaf age were randomly selected. Using the CI-340 Handheld Photosynthesis System, measurements were taken between 9:00 and 11:00 Beijing Time. To measure leaf photosynthetic performance, including net photosynthetic rate (Pn), intercellular CO_2_ concentration (Ci), stomatal conductance (Gs), and transpiration rate (Tr) under different treatments. The SPAD-502 portable chlorophyll meter (Konica Minolta Holdings) was used to measure the relative chlorophyll content (SPAD) of leaves at various growth stages of the crops. The LAI-2200C Plant Canopy Analyzer (LI-COR Biosciences) was used to measure the leaf area index (LAI).

#### 2.3.5 Production volume and its components

During the crop maturity stage, a five-point sampling method was employed to ensure the representativeness of the data. In each experimental plot, five representative sample plots (each 1 square meter in size) were selected (four located at the corners and one at the center, excluding the edge rows), with a total sampling area of 5 square meters. These plots were used to measure the number of ears, thousand-kernel weight, and grains per ear, and the theoretical yield was calculated based on these measurements. In this study, the theoretical yield was considered the final yield.

### 2.4 Model construction

#### 2.4.1 Logistic model and its improved model construction

To account for varying environmental factors such as crop growing seasons and planting dates, the cumulative temperature during winter wheat growth and development, GDD (°C·d), is analyzed as a function. Its calculation formula is as follows (Mcmaster and Wilhelm, 1997):


$$GDD=\sum_{i=1}^{n}\left(\frac{T_{max}+T_{min}}{2}-T_{b}\right)$$

Where *T_min_* represents the daily minimum temperature (°C) on day i, *T_max_* denotes the daily maximum temperature (°C) on day i, *T_b_* indicates the minimum growth temperature for wheat (°C), and *n* is the number of days.

The minimum growth temperature for wheat ranges from approximately 3 °C to 5 °C. Setting the minimum growth temperature to 0 °C yields better correlation in model predictions, thus 0 °C is adopted. Considering wheat generally ceases growth when temperatures exceed 30 °C, the maximum threshold temperature is set to 30 °C. Generally, if the daily average temperature is equal to or below the base temperature, no degree days are accumulated. When the average temperature exceeds the threshold temperature, it is recorded as 30 °C (Mahbod et al., 2014).

Logistic and improved Logistic models with GDD as the independent variable were employed to analyze regional variations in plant height H (cm), dry matter accumulation DMA (kg/ha), and leaf area index LAI:


$$Y_{1}=\frac{L}{1+e^{-aGDD+b}}$$

$$Y_{2}=\frac{C}{1+e^{dGDD^{2}+fGDD+g}}$$

Where *Y_1_* and *Y_2_* both physiological growth traits (in this study, *Y_1_* denotes plant height and dry matter accumulation; *Y_2_* denotes leaf area index), GDD denotes the cumulative heat units during the growing season (measured in degree days; in this study, cumulative temperature values were used), and *L*, *a*, *b*, *c*, *d*, *f*, g are equation coefficients.


$$\;Y_{1}^{\prime }=\frac{aLe^{-aGDD+b}}{{\left(1+e^{-aGDD+b}\right)}^{2}}$$

$$\;Y_{1}^{\prime \prime }=\frac{-a^{2}Le^{-aGDD+b}\left(1+e^{-aGDD+b}\right)}{{\left(1+e^{-aGDD+b}\right)}^{3}}$$

Taking the first derivative of equation *Y_1_* yields the growth rate equation: $Y_{1}^{\prime }$; Taking the first derivative of the growth rate equation and setting it to zero determines the maximum growth rate V_1_ and the required accumulated temperature GDD_1_ to reach this maximum; Taking the second derivative of the growth rate equation and setting it to zero yields the two inflection points GDD_2_ and GDD_3_ on the growth curve. From sowing to GDD_2_ and from GDD_3_ to winter wheat harvest, the growth rate is in the gradually increasing phase and the slowly increasing phase, respectively. The growth rate is in the rapid growth phase between GDD_2_ and GDD_3_

Differentiating the equation *Y_2_* yields the rate equation for LAI change:


$$Y_{2}^{\prime }$$

$$\;Y_{2}^{\prime }=\frac{-ce^{dGDD^{2}+fGDD+g}\left(f+2d\right)}{{\left(1+e^{dGDD^{2}+fGDD+g}\right)}^{2}}$$

Setting $Y_{2}^{\prime }$ to zero and solving yields the accumulated temperature C_xmax_ required to reach maximum LAI. Differentiating equation $Y_{2}^{\prime }$ yields the acceleration equation for LAI change. Setting this to zero and solving numerically over the interval 0 to C_xmax_ gives the accumulated temperature C_xinf_ at which LAI reaches its maximum expansion rate. Substituting this into equation $Y_{2}^{\prime }$ yields the maximum LAI expansion rate C_Rmax_

#### 2.4.2 Model evaluation

Using the Logistic model and the improved Logistic model to simulate the physiological growth indicators of winter wheat under the WFPSR for 2023-2025, and to validate and evaluate the calibrated models, Evaluation metrics include: R^2^ (coefficient of determination); RMSE (root mean square error); NRMSE (normalized root mean square error); d (index of agreement).


$$R^{2}={\left(\frac{\sum_{i=1}^{n}\left(P_{i}-\bar{P}\right)\left(O_{i}-\bar{O}\right)}{\sqrt{\sum_{i=1}^{n}{\left(P_{i}-\bar{P}\right)}^{2}\sum_{i=1}^{n}{\left(O_{i}-\bar{O}\right)}^{2}}}\right)}^{2}$$

$$RMSE=\sqrt{\frac{\sum_{i=1}^{n}{\left(P_{i}-O_{i}\right)}^{2}}{n}}$$

$$nRMSE=\frac{\sqrt{\frac{\sum_{i=1}^{n}{\left(P_{i}-O_{i}\right)}^{2}}{n}}}{\bar{O}}$$

$$d=1-\frac{\sum_{i=1}^{n}{\left(P_{i}-O_{i}\right)}^{2}}{\sum_{i=1}^{n}{\left(\left|P_{i}-\bar{O}\right|+\left|O_{i}-\bar{O}\right|\right)}^{2}}$$

*P_i_* and *O_i_* represent the predicted and measured values of the parameters, respectively. *n* denotes the number of measurements. $\bar{O}$ and $\bar{P}$ are the mean values of the measured and predicted values, respectively.

### 2.5 Data processing

In this study, Microsoft Excel 2019 was used to collect and preliminarily organize the experimental data. To ensure the robustness of traditional univariate statistical inference, two-way analysis of variance and *post-hoc* comparisons were performed using IBM SPSS Statistics 27.0. Prior to performing analysis of variance (ANOVA), the Shapiro-Wilk test was used to assess the normality of the data distribution, and Levene’s test was used to confirm the homogeneity of variances across groups. For data that met the assumptions, Duncan’s modified *post-hoc* multiple comparison test was performed (P< 0.05). Given its strengths in complex numerical calculations and nonlinear fitting, MATLAB 2022 software was used to construct a logistic growth model under different real-time water-nutrient-pesticide control conditions. To enable efficient processing and high-quality visualization of multivariate statistical data, Origin 2021 software was used to perform PCA dimensionality reduction analysis and correlation analysis, as well as to plot diagrams of various growth indicators, temperature, precipitation, and crop growth models. To accurately represent geospatial information, ArcGIS 10.8 software was used to create a general map of the study area.

## 3 Results

### 3.1 Relative chlorophyll values in winter wheat leaves

**Figure 3:**
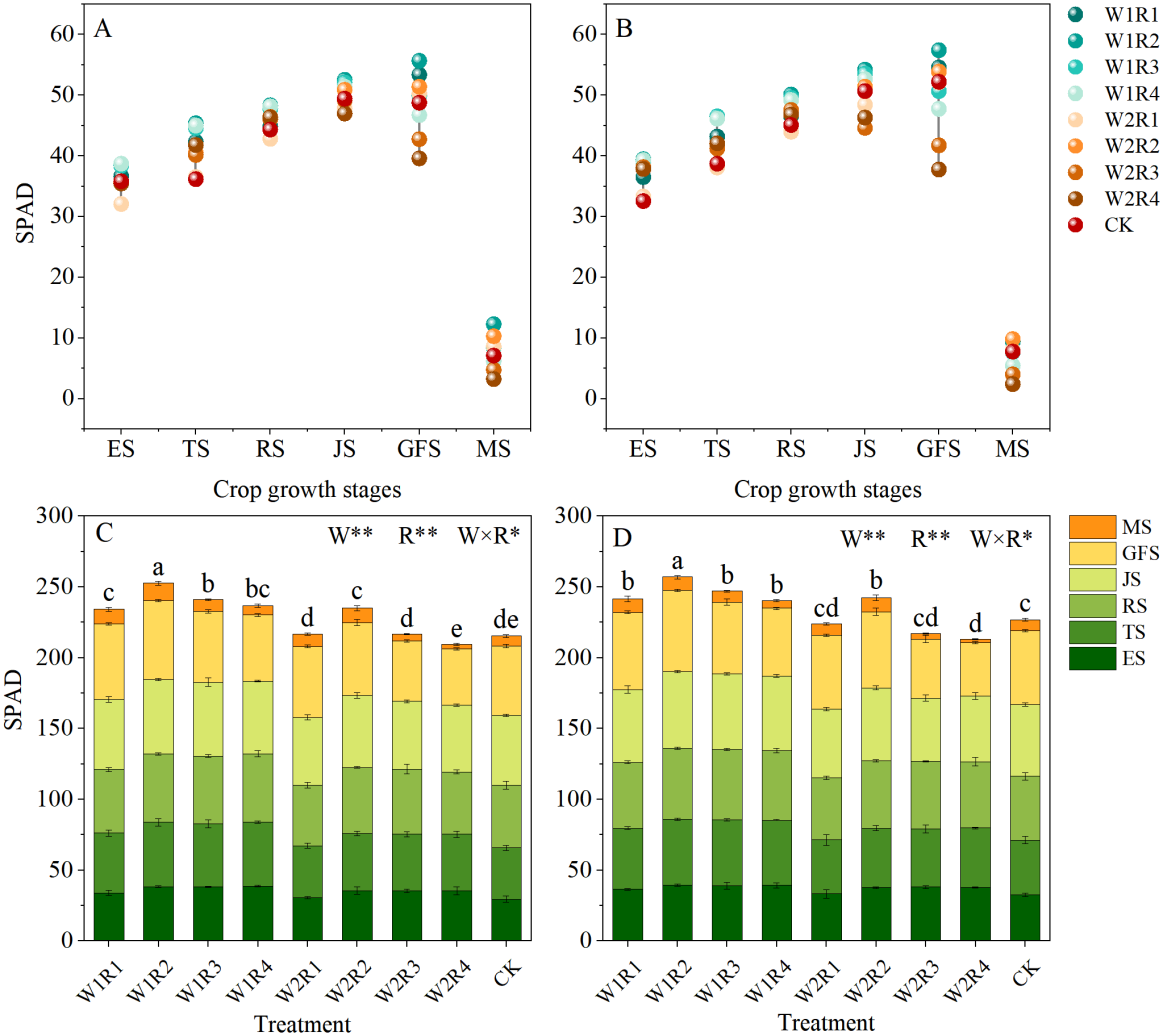
Characteristics of changes in relative chlorophyll values in winter wheat leaves during the 2023-2025. **(A, B)** show changes in leaf SPAD values of winter wheat over the growth stages for the 2023–2024 and 2024-2025, respectively; **(C, D)** show bar charts of the analysis of variance for leaf SPAD values across growth stages in winter wheat in the 2023–2024 and 2024-2025, respectively. Lowercase letters indicate significant differences among treatments (p< 0.05); W: real-time irrigation level; R: fertilizer-pesticide treatment; *p< 0.05, **p< 0.01.

From 2023-2025, the relative chlorophyll content (SPAD) of winter wheat leaves under the WFPSR showed a trend of initially increasing and then decreasing as the growing stages progressed. SPAD values increased slowly from the emergence stage to the jointing stage, reached a maximum during the grain-filling stage, and then declined rapidly at maturity (**Figures 3A, B**). Analysis of variance revealed that, between these two growing seasons, the real-time irrigation level (W), the fertilizer-pesticide treatment (R), and their interaction (W×R) all had a significant effect on the SPAD values of winter wheat leaves (p< 0.05) (**Figures 3C, D**). Under the same fertilizer-pesticide treatment, the leaf SPAD values for the W1 irrigation treatment were significantly higher than those for the W2 treatment. At the same irrigation level, the leaf SPAD values in the R2 fertilizer-pesticide treatment were notably higher; at the W1 irrigation level, they were 4.40% and 6.79% higher than those in the R3 and R4 treatments, respectively, and at the W2 irrigation level, the difference compared to the R3 and R4 treatments widened further. In 2023-2024, the SPAD values for treatments W1R2, W1R3, W1R4, and W2R2 were significantly higher than those of the CK by 17.31%, 11.96%, 9.99%, and 9.17%, respectively; but remaining treatments effect found non-significant. In 2024-2025, the differences among treatments were largely consistent with those of the previous growing season, while the SPAD value for treatment W2R4 decreased by 6.07% compared to CK.

### 3.2 Photosynthetic characteristics of winter wheat leaves

**Figure 4:**
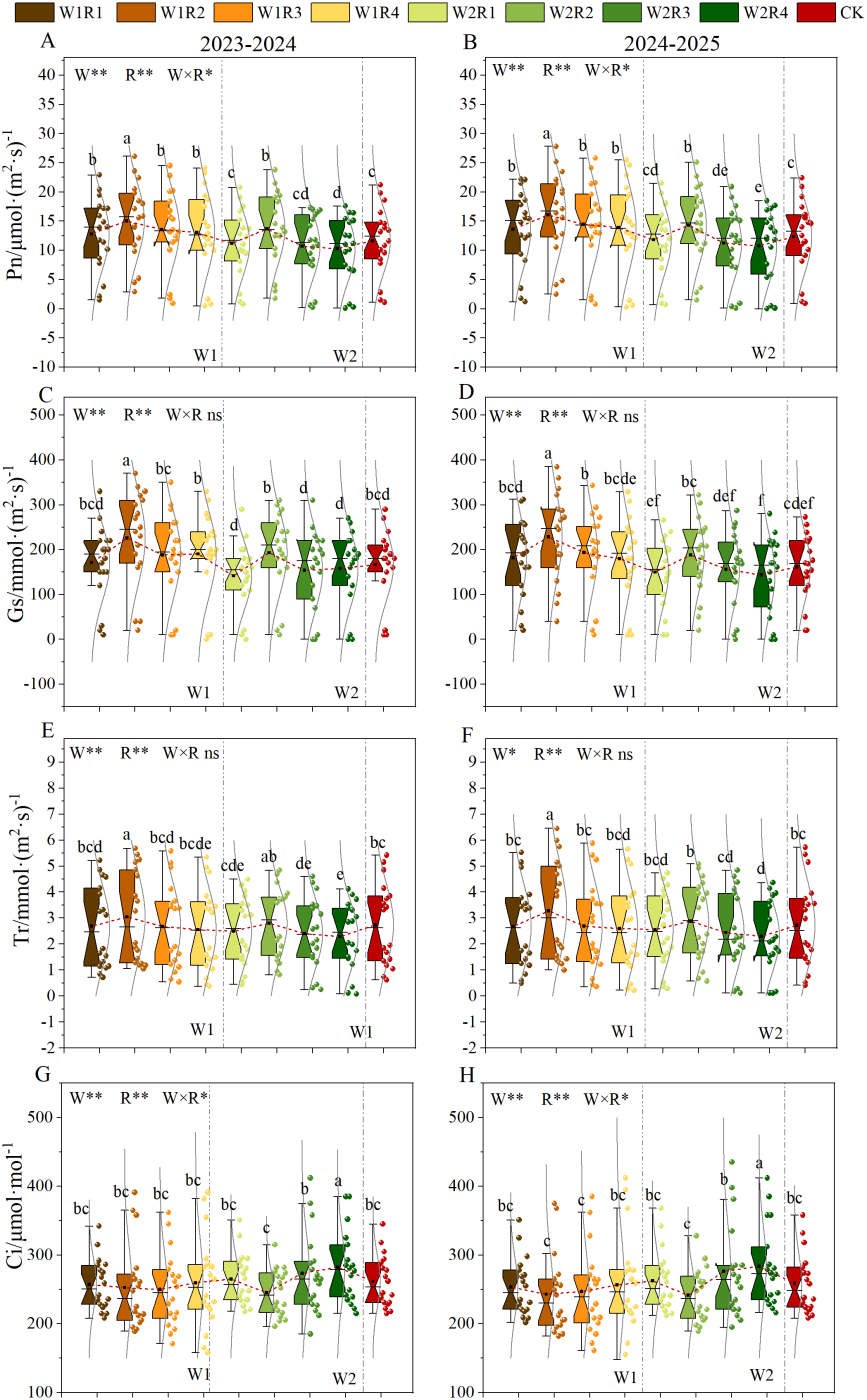
Effects of WFPSR on the photosynthetic characteristics of winter wheat leaves during the 2023-2025. **(A, C, E, G)** show the significance analysis of Pn, Gs, Tr, and Ci in 2023-2024, respectively; **(B, D, F, H)** show the significance analysis of Pn, Gs, Tr, and Ci in 2024-2025, respectively. Lowercase letters indicate significant differences among treatments (p< 0.05); W: real-time irrigation level; R: fertilizer-pesticide treatment; *p< 0.05, **p< 0.01.

During the 2023-2025, both the real-time irrigation level (W) and the fertilizer-pesticide treatment (R) had significant effects (p< 0.05) on the net photosynthetic rate (Pn), stomatal conductance (Gs), and transpiration rate (Tr) of wheat leaves (**Figures 4A–F**). The interaction between real-time irrigation levels and fertilizer-pesticide treatments (W×R) significantly affected Pn (p< 0.05) but had no significant effect on Gs and Tr. Under the same fertilizer-pesticide treatment, as real-time irrigation levels increased, the values of Pn, Gs, and Tr in winter wheat leaves showed an overall downward trend, with W1 > W2 observed in 2023–2024 and 2024-2025. Under the same real-time irrigation level, the leaf Pn, Gs, and Tr values of the 33% reduced-pesticide treatment combined with microbial biofertilizer were significantly superior to those of other reduced-pesticide treatments, showing increases of 23.14%, 21.95%, and 17.24%, respectively, compared to the R4 treatment. The differences among the R1, R3, and R4 treatments were minor, with leaf Pn, Gs, and Tr values showing essentially no significant differences. Compared with CK, the W1R2 treatment showed the most significant differences in Pn, Gs, and Tr during these two seasons.

In 2023-2025, the real-time irrigation level (W) and fertilizer-pesticide treatment (R), as well as their interaction (W×R), significantly affected the intercellular CO_2_ concentration (Ci) in winter wheat leaves (**Figures 4G, H**). Under the same fertilizer-pesticide treatment, the leaf Ci values in the W2 treatment were generally significantly higher than those in the W1 treatment in 2023-2025. Under the W1 real-time irrigation regime, there were no significant differences among the fertilizer-pesticide treatments, but the leaf Ci in the W1R2 treatment remained at a low level. Under the W2 real-time irrigation level, leaf Ci showed a significant upward trend as the reduction in pesticide application increased; leaf Ci in the W2R2 treatment was 19.55% lower than that in the W2R4 treatment. During the 2023-2025, the leaf Ci of the W1R2 treatment showed a significant difference compared to the CK treatment, decreasing by 4.76%.

### 3.3 Leaf area index of winter wheat

#### 3.3.1 The response patterns of the leaf area index in winter wheat

**Figure 5:**
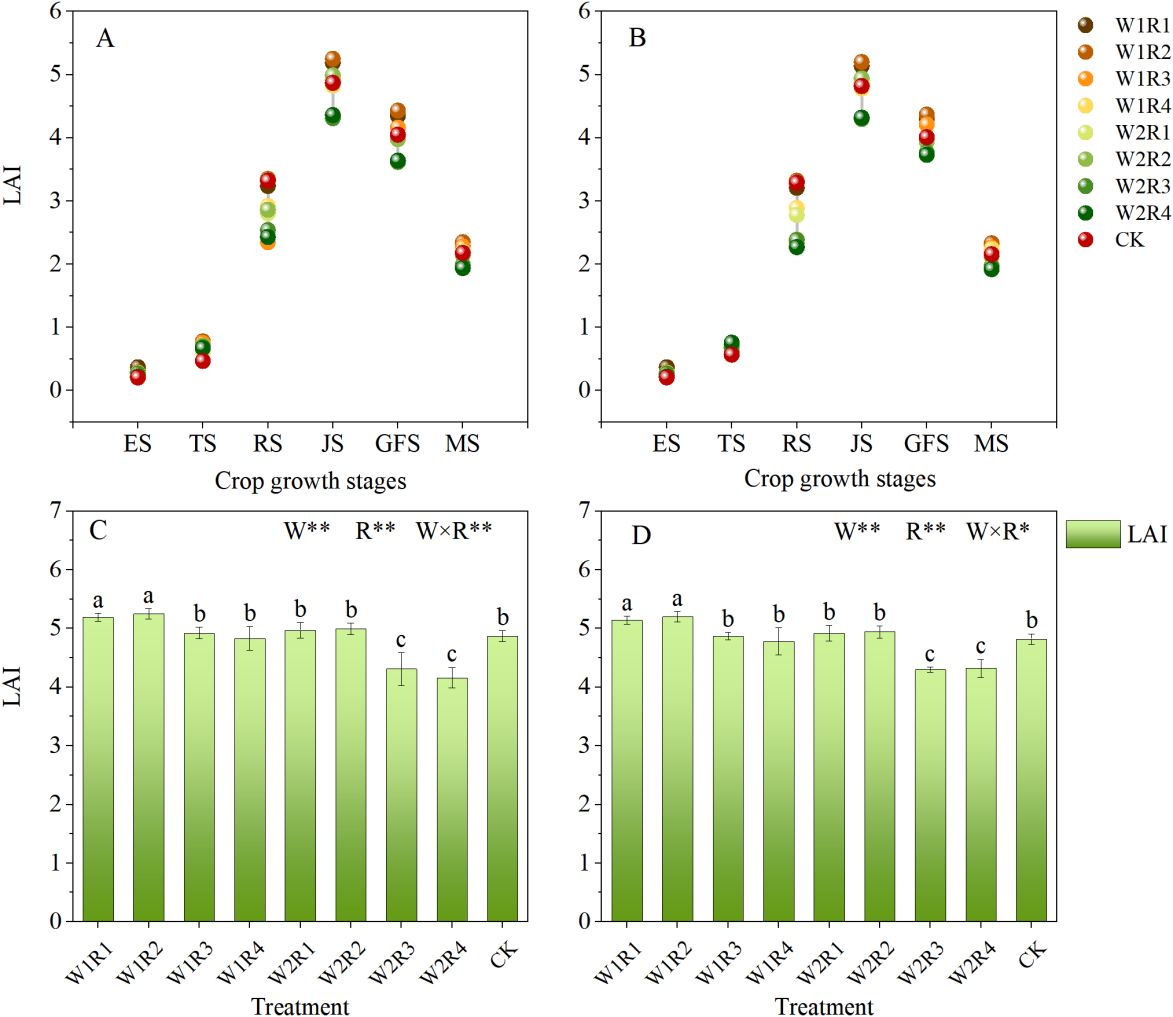
Characteristics of changes in leaf area index during the growth stages of winter wheat in 2023-2025. **(A, B)** show the changes in leaf area index of winter wheat as the growth stages progressed in 2023–2024 and 2024-2025, respectively. **(C, D)** show the histograms of the analysis of variance (ANOVA) for leaf area index at the jointing stage in 2023–2024 and 2024-2025, respectively; Lowercase letters indicate significant differences among treatments (p< 0.05); W, real-time irrigation level; R, fertilizer-pesticide treatment; *p< 0.05, **p< 0.01.

Under the WFPSR, the leaf area index (LAI) of winter wheat exhibited a single-peak trend—rising initially and then declining—as the growing stages progressed during the 2023-2025. During the seedling and tillering stages, the LAI of winter wheat increased gradually, with significant differences emerging from the regreening stage onward; by the jointing stage, the LAI reached its peak. As the growing stages progressed, the LAI of winter wheat gradually decreased, and the differences among treatments gradually narrowed (**Figures 5A, B**). During the two seasons, both the level of real-time irrigation (W) and fertilizer-pesticide treatments (R), as well as their interaction (W×R), significantly influenced the leaf area index of winter wheat during the jointing stage (P< 0.05) (**Figures 5C, D**). Under the same fertilizer-pesticide treatment regimen, the leaf area index (LAI) values for the W1 treatment were generally significantly higher than those for the W2 treatment. Under the same real-time irrigation level, the leaf area index of winter wheat initially increased slightly with reduced pesticide application and the addition of microbial biofertilizer, but showed a significant decrease when pesticide application was reduced by more than 33% even with the application of microbial biofertilizer. During the 2023-2025, the LAI at the jointing stage in the W1R2 and W2R2 treatments increased by 8.74% and 14.40%, respectively, compared to the W1R4 and W2R4 treatments. The LAI at the jointing stage in the CK showed no significant difference compared to treatments such as W1R4 and W2R1, but the W1R2 treatment was significantly higher than CK by 7.84%. These results indicate that, compared to conventional irrigation and fertilizer-pesticide application, appropriate WFPSR can significantly promote the expansion of winter wheat leaf area.

#### 3.3.2 Temporal dynamics in winter wheat leaf area index based on the improved logistic model

**Figure 6:**
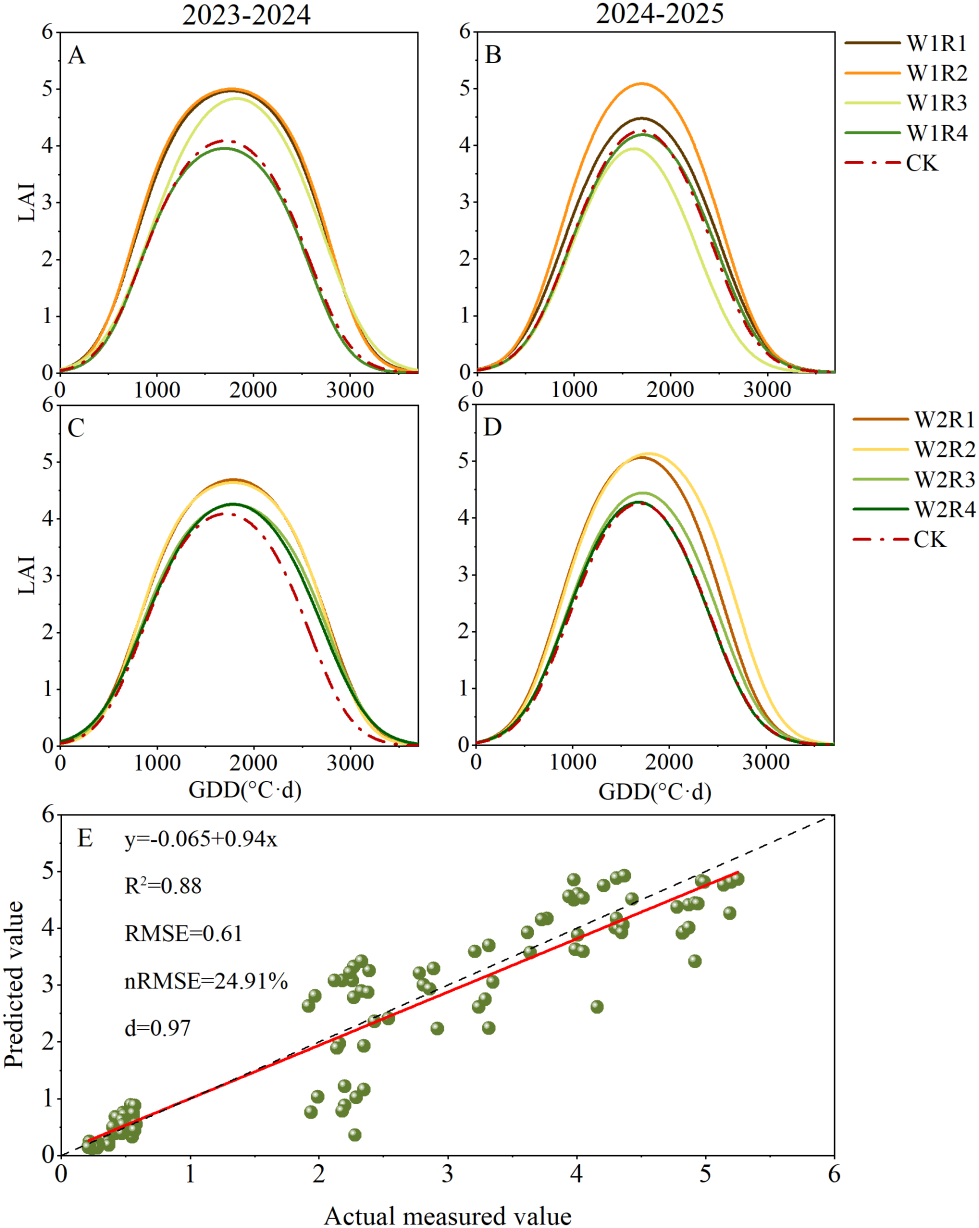
Fitting and validation results of the modified logistic model for winter wheat leaf area index in 2023-2025. **(A, C)** show the fitting results of the modified logistic model for winter wheat leaf area index in 2023-2024. **(B, D)** show the fitting results of the modified logistic model for winter wheat leaf area index in 2024-2025. **(E)** Validation results of the modified logistic model for winter wheat leaf area index for 2023-2025.

An improved Logistic model was used to fit the dynamic changes in the leaf area index of winter wheat for 2023–2024 and 2024-2025 (**Figures 6A–D**). The model validation results were satisfactory (**Figure 6E**), indicating that the model can accurately simulate the dynamic characteristics of the winter wheat leaf area index as a function of cumulative temperature.

The temporal dynamic parameters of winter wheat LAI were calculated based on the fitting equations for 2023–2024 and 2024-2025. The temporal dynamic parameters of winter wheat LAI over the two seasons were significantly influenced by WFPSR. Under the same fertilizer-pesticide treatment, the accumulated temperature required for winter wheat to reach the maximum LAI expansion rate (C_xinf_) and the accumulated temperature required to reach peak LAI (C_xmax_) showed a pattern of W1< W2 over the two seasons, while the maximum expansion rate (C_Rmax_) showed a pattern of W1 > W2. Under the same irrigation level, as the proportion of pesticide reduction increased, C_xinf_ and C_xmax_ of winter wheat exhibited a delayed trend, and C_Rmax_ also gradually decreased. During the two-year observation period, the 33% pesticide reduction combined with microbial biofertilizer treatment maintained the optimal growth dynamics; in the high-pesticide-reduction R4 treatment, _Cxinf_ was delayed by 6.80% compared to R2. Meanwhile, the C_Rmax_ of the R2 treatment was significantly higher than that of the R4 treatments by 19.15%. Compared with the CK treatment, the W1R2 treatment reached its maximum expansion rate significantly earlier and the maximum expansion rate C_Rmax_ increased by 25.27%.

### 3.4 Plant height of winter wheat

#### 3.4.1 The response patterns of winter wheat plant height

**Figure 7:**
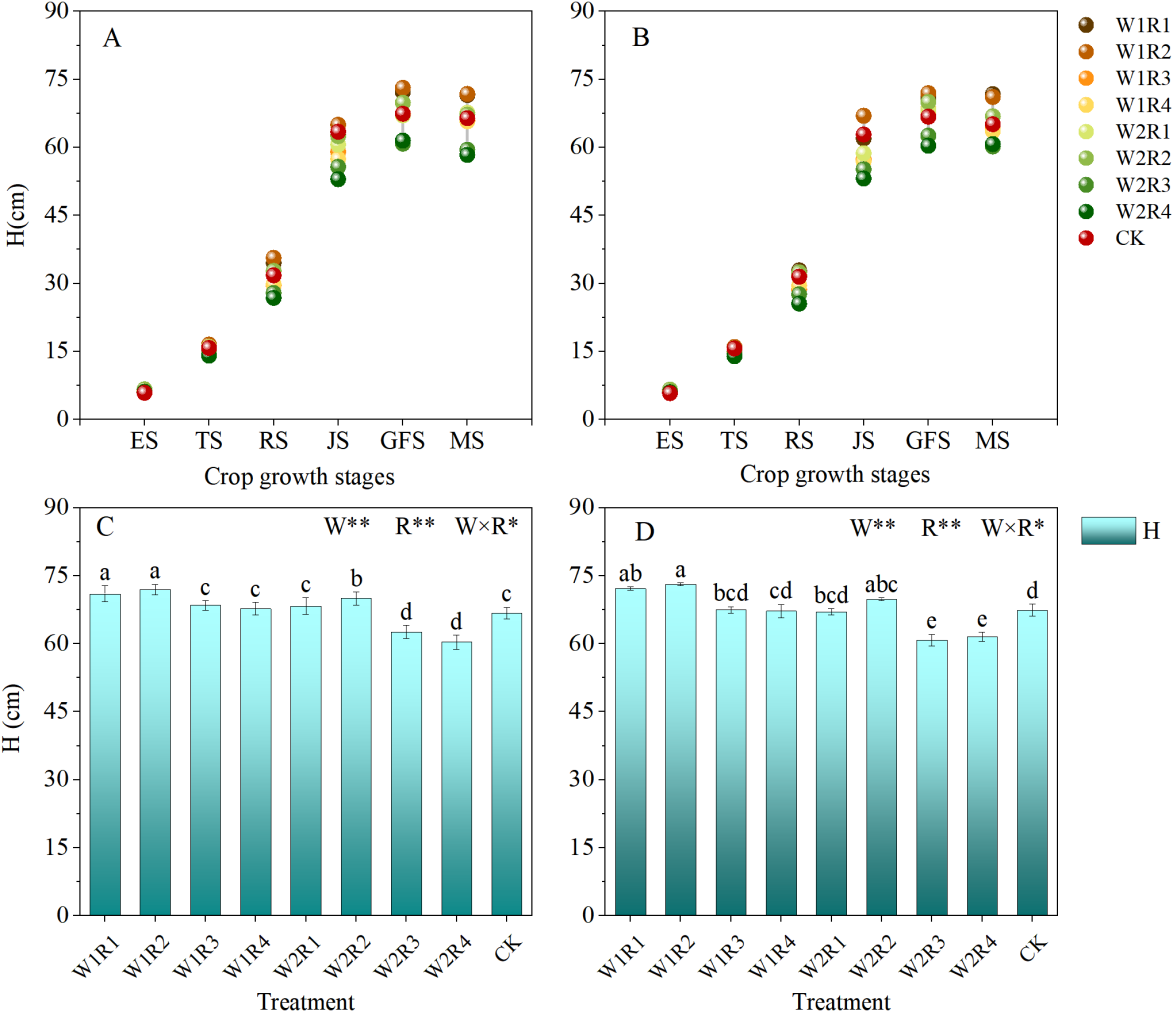
Changes in winter wheat plant height during the growing seasons in 2023-2025. **(A, B)** show changes in plant height as the growing season progressed in 2023–2024 and 2024-2025, respectively. **(C, D)** show bar charts of the analysis of variance (ANOVA) for plant height during the grain-filling stage in 2023–2024 and 2024-2025, respectively; Lowercase letters indicate significant differences among treatments (p< 0.05); W, real-time irrigation level; R, fertilizer-pesticide treatment; *p< 0.05, **p< 0.01.

During the 2023-2025, the trend in winter wheat plant height (H) as a function of cumulative temperature was generally consistent under WFPSR. From emergence to the regreening stage, plant height increased gradually, with significant differences beginning to emerge during the regreening stage. A rapid elongation phase was observed between the heading and grain-filling stages; thereafter, as the spikes matured, plant height tended to stabilize or decline slightly (**Figures 7A, B**). Different irrigation levels (W) and fertilizer-pesticide treatments (R), as well as their interaction (W×R), all significantly affected the plant height of winter wheat during the grain-filling stage (P< 0.05) (**Figures 7C, D**). Under the same fertilizer-pesticide treatment, winter wheat plant height over the two seasons showed a pattern of W1 > W2, with an average difference of 4.83 cm. Under the same irrigation level, plant height in the R2 treatment was significantly higher than in other treatments, exceeding the R1, R3, and R4 treatment groups by 2.29%, 9.88%, and 10.95%, respectively. During the 2023–2025 growing seasons, the W1R2 treatment showed a significant advantage in plant height compared to CK, while the W2R3 and W2R4 treatments exhibited stunted growth.

#### 3.4.2 Temporal dynamics in winter wheat plant height based on the logistic model

**Figure 8:**
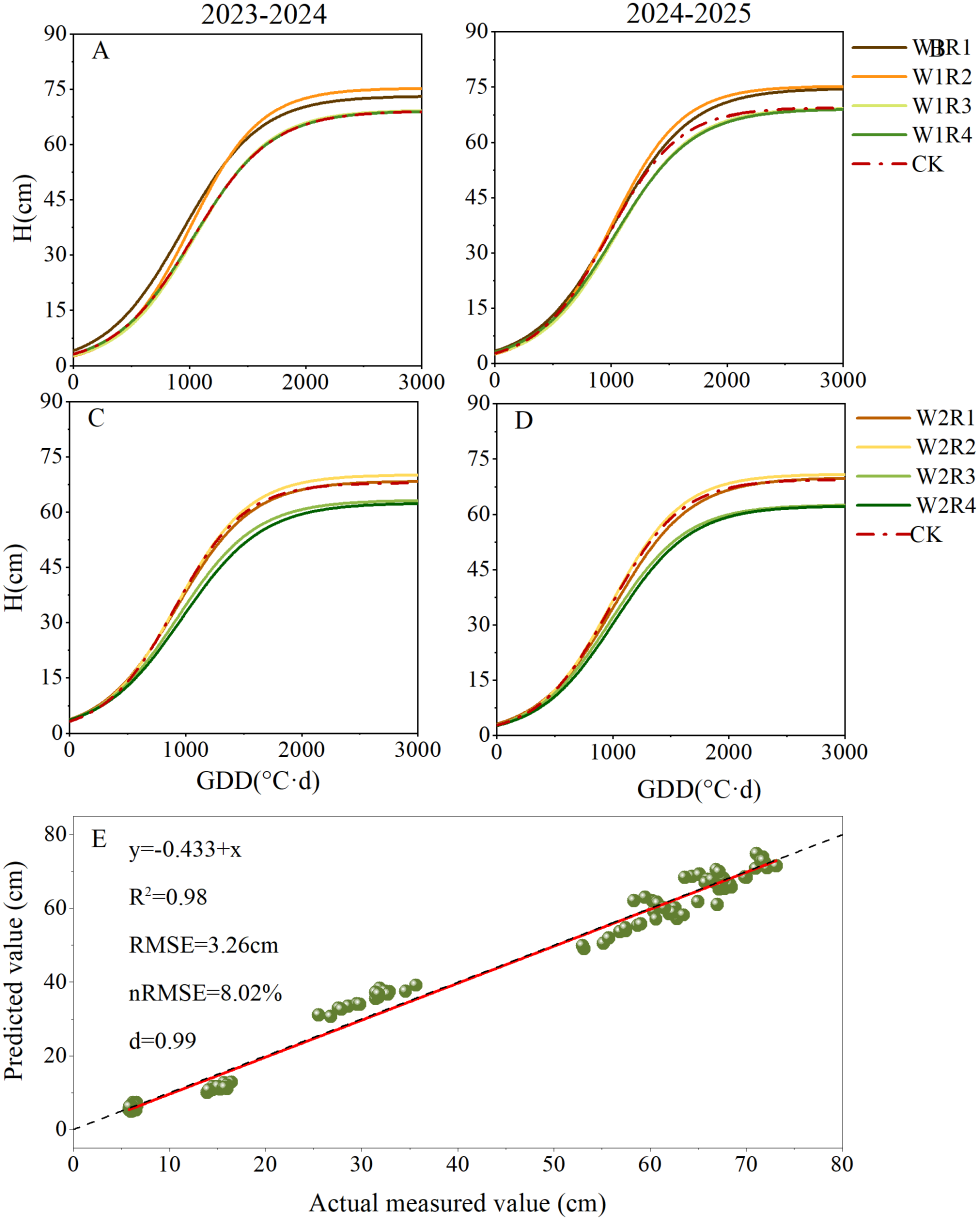
Fitting and validation results of the Logistic model for winter wheat plant height in 2023-2025. **(A, C)** show the fitting results of the Logistic model for plant height in 2023-2024. **(B, D)** show the fitting results of the Logistic model for plant height in 2024-2025. **(E)** shows the validation results of the Logistic model for winter wheat plant height in 2023-2025.

A logistic model was used to fit the dynamic changes in winter wheat plant height (H) for 2023–2024 and 2024-2025 (**Figures 8A–D**). Validation results (**Figure 8E**) indicate that the model accurately simulates the dynamic growth patterns of winter wheat plant height as accumulated temperature increases.

The parameters describing the temporal dynamics of winter wheat plant height were calculated based on the fitting equations for 2023-2025. Under the same fertilizer and pesticide treatment, the maximum growth rate of plant height (V_1_) generally followed the pattern W1 > W2, and the duration of the rapid growth phase for plant height under the W1 irrigation level was 16.81 °C·d longer than that under the W2 treatment. Under the same irrigation regime, the maximum growth rate of plant height generally showed a trend of first increasing and then decreasing with increasing fertilizer reduction, peaking at R2. Over the two seasons, the maximum growth rate at R2 was significantly higher than that at R4 by 20.62%, and the duration of the rapid growth phase for low-reduction treatments such as R1 and R2 was 23.91 °C·d shorter than treatments such as R3 and R4. Compared to CK, treatments such as W1R2—which applied low-concentration chemical fertilizer in combination with microbial fertilizer—increased the maximum growth rate of plant height and shortened the duration of the rapid growth phase to varying degrees, thereby demonstrating higher growth efficiency.

### 3.5 Dry matter accumulation in winter wheat

#### 3.5.1 The response patterns of dry matter accumulation in winter wheat

**Figure 9:**
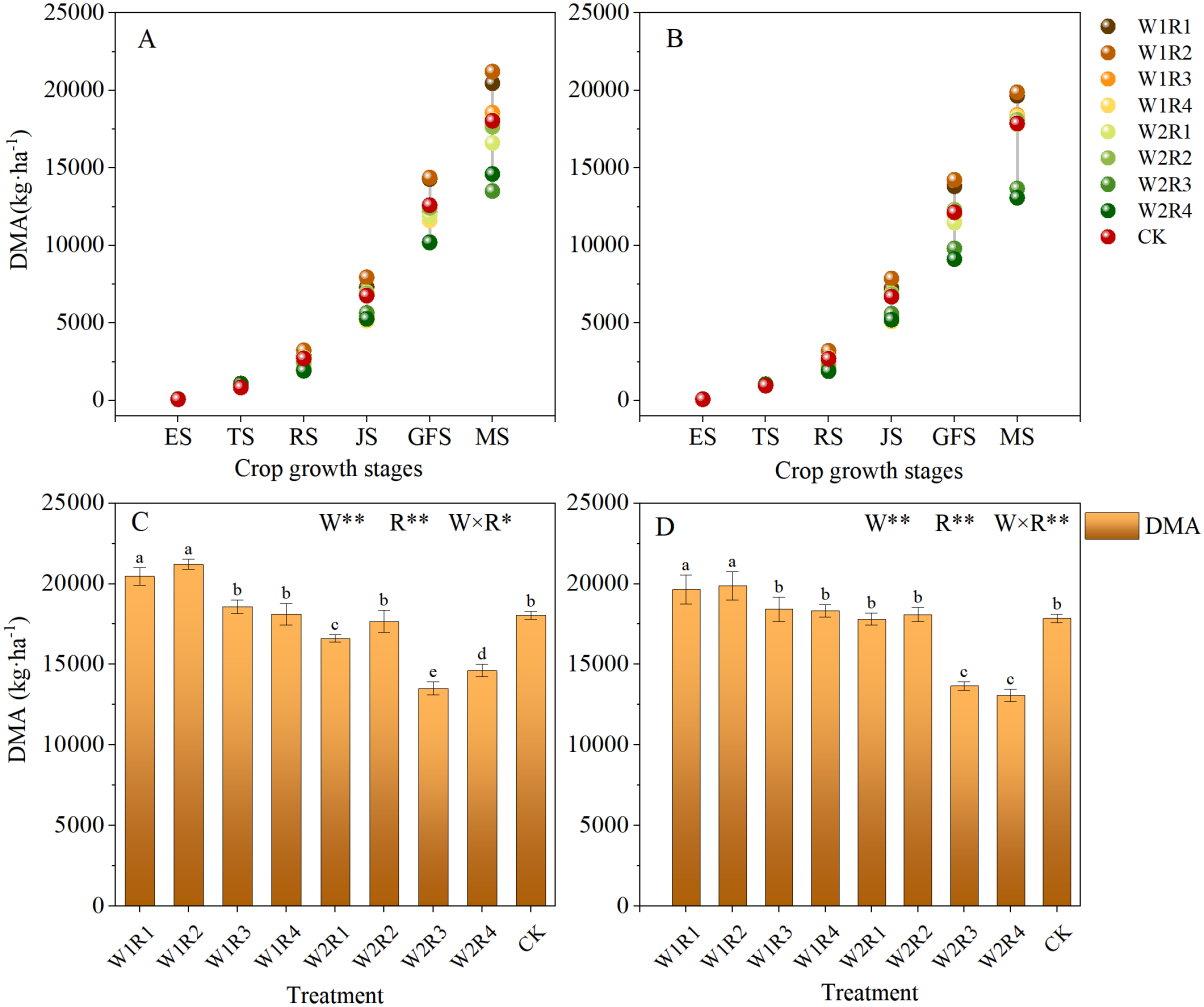
Characteristics of dry matter accumulation during the growth stages of winter wheat in 2023-2025: **(A, B)** show the changes in dry matter accumulation of winter wheat as the growth stages progressed in 2023–2024 and 2024-2025, respectively; **(C, D)** show the bar charts of analysis of variance (ANOVA) for dry matter accumulation at maturity in 2023–2024 and 2024-202, respectively; Lowercase letters indicate significant differences among treatments (p< 0.05); W, real-time irrigation level; R, fertilizer-pesticide treatment; *p< 0.05, **p< 0.01.

During the 2023–2025 growing seasons, the trends in wheat dry matter accumulation (DMA) as the growing season progressed were generally consistent under WFPSR. From the emergence stage to the tillering stage, dry matter accumulation proceeded slowly. From the regreening stage to the grain-filling stage, the accumulation rate first accelerated and then slowed again; starting from the jointing stage, significant differences began to emerge among the treatments, eventually stabilizing at the maturity stage, exhibiting a typical “slow-fast-slow” S-shaped growth curve (**Figures 9A, B**). Real-time irrigation levels (W) and fertilizer-pesticide treatments (R), as well as their interaction (W×R), all significantly influenced dry matter accumulation during the crop’s maturation period (P< 0.05) (**Figures 9C, D**). During these two growing seasons, under the same fertilizer-pesticide treatment, the W1 treatment yielded significantly higher dry matter accumulation than the W2 treatment, with the former exceeding the latter by 23.6%. At the same irrigation level, the pesticide reduction ratio in the fertilizer-pesticide treatment exhibited a significant threshold effect on dry matter accumulation; when microbial biofertilizers are applied and the pesticide reduction ratio exceeded 33%, wheat DMA decreased significantly. The DMA at maturity for R2 was relatively the highest among all treatments, with a smaller difference compared to R1; both were significantly higher than the R4 treatment. In 2023-2025, the DMA during the maturity stage of the W1R2 treatment was the highest among all combinations, showing a significant increase of 11.28% compared to CK, while the W2R4 treatment showed a significant decrease of 26.77% compared to CK.

#### 3.5.2 Temporal dynamics of dry matter accumulation in winter wheat based on the logistic model

**Figure 10:**
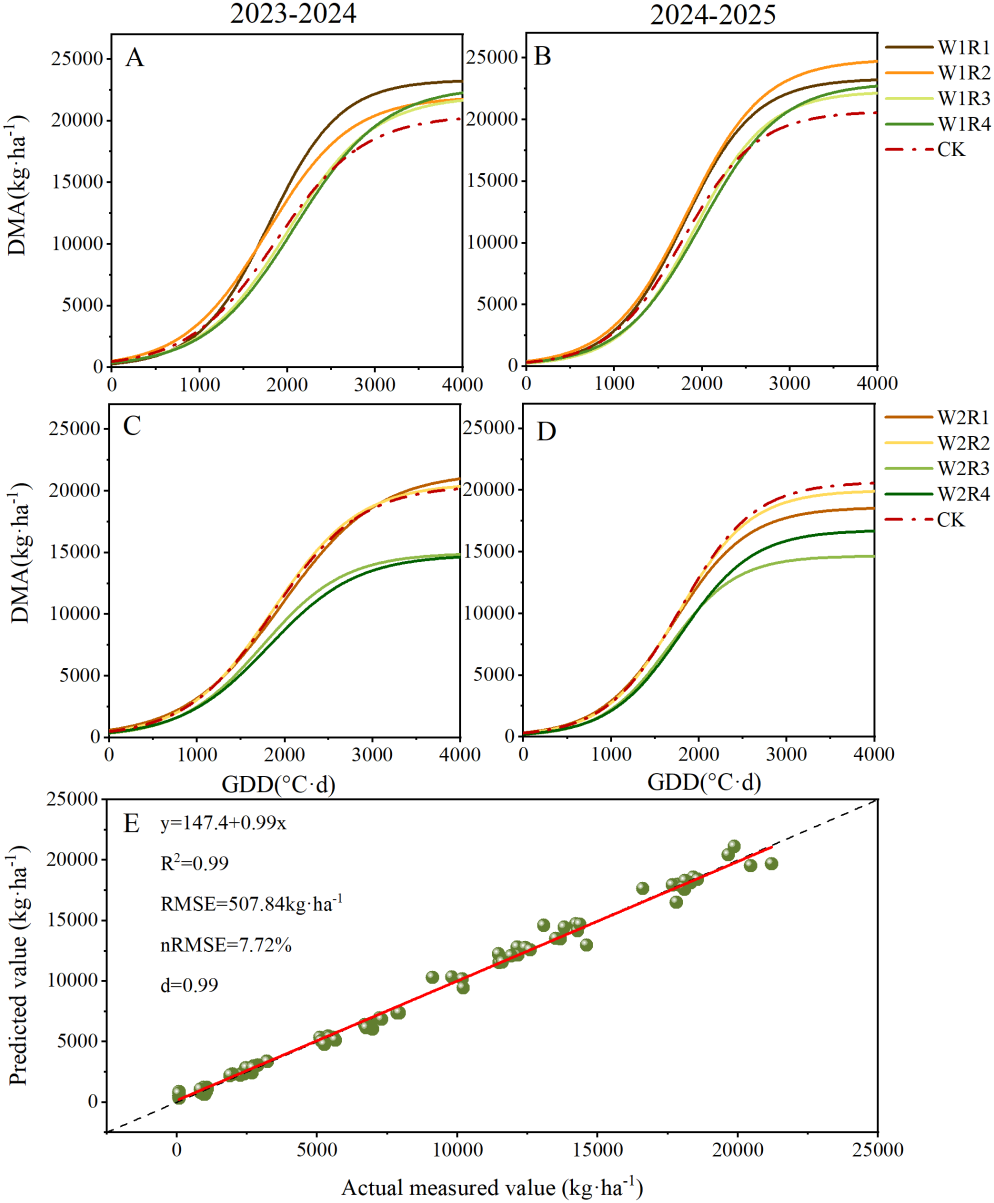
Fitting and validation results of the logistic model for dry matter accumulation in winter wheat for 2023-2025. **(A, C)** show the fitting results of the Logistic model for dry matter accumulation in 2023-2024. **(B, D)** show the fitting results of the Logistic model for dry matter accumulation in 2024-2025. **(E)** Validation results of the Logistic model for dry matter accumulation in winter wheat for 2023-2025.

A logistic model was used to fit the dynamic changes in dry matter accumulation (DMA) of winter wheat in 2023 and 2024 (**Figures 10A–D**). The model fit was well validated (**Figure 10E**), and the model was able to accurately simulate the dynamic characteristics of winter wheat dry matter accumulation as a function of accumulated temperature.

The dynamic parameters of winter wheat DMA were calculated based on the fitted equations for 2023 and 2024. Under the same fertilizer and pesticide treatment, the maximum DMA growth rates (V1) for treatments W1 and W2 were 14.09 kg·ha^-1^·°C·d and 9.94 kg·ha^-1^·°C·d, respectively, with the former being 29.44% higher than the latter. The duration of the rapid growth phase for DMA in the W1 treatment was 7.66 °C·d longer than that in the W2 treatment. Under the same real-time irrigation level, the duration of the rapid growth phase for dry matter accumulation in the R2 treatment was 35.08 °C·d shorter than that in the R4 treatment. The maximum growth rate in the R2 treatment was 16.43% higher than that in the R4 treatment. Compared with the CK, the maximum dry matter growth rate of the W1R2 treatment increased significantly by 14.09%, and the duration of the rapid growth phase was shortened by 6.78 °C·d.

### 3.6 Quantitative analysis of the relationship between winter wheat yield and physiological growth indices

#### 3.6.1 Principal component analysis of physiological growth indices of winter wheat and their correlation with yield

**Figure 11:**
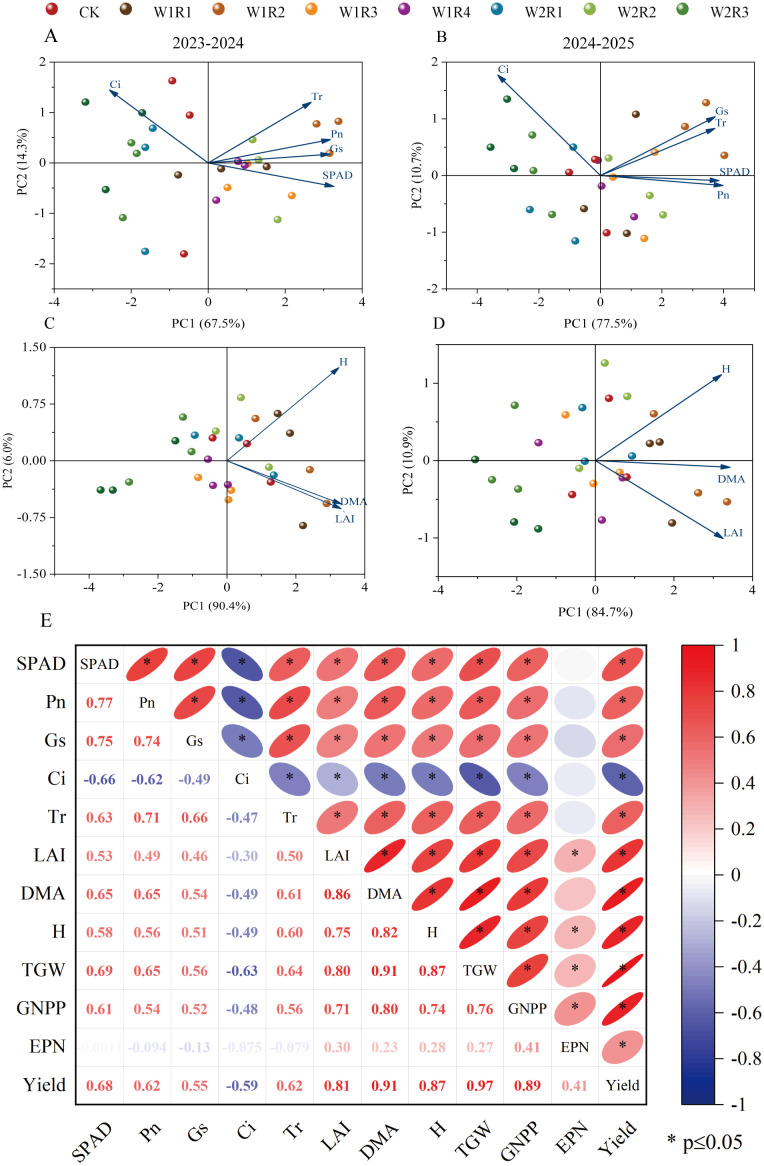
PCA dimension reduction analysis of various physiological growth indicators for winter wheat in 2023–2025 and the yield correlation matrix. **(A, B)** show the principal component analysis of photosynthetic physiological indicators for winter wheat in 2023–2024 and 2024-2025. **(C, D)** show the principal component analysis of population structure indicators for winter wheat in 2023–2024 and 2024-2025. **(E)** presents the correlation matrix analysis between winter wheat physiological growth indicators and yield and its components; darker colors indicate higher correlations between indicators, red indicates positive correlations, blue indicates negative correlations, and * denotes significant correlations (p ≤ 0.05).

Based on experimental data from 2023-2025, principal component analysis was performed on five leaf photosynthetic physiological indicators and three population structure indicators of winter wheat under WFPSR. A composite plot of the loadings and scores is shown in **Figure 11**. Analysis of wheat leaf photosynthetic physiological indicators for two seasons (**Figures 11A, B**) shows that the cumulative variance explained by the first two principal components exceeded 81.8%, effectively summarizing the majority of the sample information. In 2023, Principal Component 1 (PC1) and Principal Component 2 (PC2) accounted for 67.5% and 14.3% of the original information, respectively. In 2024, they accounted for 77.5% and 10.7%, respectively. Analysis of population structure indices during the same period (**Figures 11C, D**) revealed even higher information concentration: in 2023, PC1 and PC2 explained 90.4% and 6.0% of the total variation, respectively, while in 2024, these figures were 84.7% and 10.9%. Furthermore, analysis of the angles and projection distances between the vectors of the various leaf photosynthetic physiological indices in **Figures 11A, B** revealed that the angles between Pn, Gs, Tr, and SPAD were small, indicating a significant positive correlation; conversely, the vector direction of Ci formed obtuse angles with the other four indices, suggesting a significant negative correlation between Ci and these indices. Regarding population structure indices, the vectors of H, DMA, and LAI form acute angles with one another, indicating a close positive synergy among all population structure indices. By observing the projection magnitudes of each physiological and growth indicator along the positive half-axis of PC1 in **Figure 11**, it can be seen that the treatment combining the 80%θ*_fc_*-60%θ*_fc_* real-time irrigation level with 33% reduced pesticide application and microbial biofertilizer (W1R2) exhibited significantly greater projection variance along the PC1 direction than the CK and other WFPSR treatments. This indicates that the W1R2 treatment group carries the greatest amount of information across all physiological growth indicators, thereby achieving a higher comprehensive evaluation score in the PCA dimensionality reduction analysis.

To evaluate the correlations between various physiological growth indices of winter wheat and its yield and yield components under WFPSR, this study constructed a correlation matrix based on data from 2023-2024 (**Figure 11E**). Most physiological growth indicators of winter wheat showed a significant positive correlation with final yield (Yield) (P< 0.05). Regarding yield components, under WFPSR, thousand-grain weight (TGW) and number of grains per panicle (NGPP) were the primary factors influencing final yield, with significant correlation coefficients of 0.97 and 0.89, respectively. TGW showed significant positive correlations with DMA, SPAD, and LAI, with correlation coefficients of 0.91, 0.69, and 0.80, respectively. In contrast, the positive correlation between the effective panicle number (EPN) and yield was relatively weak among all significant indicators, and EPN showed no significant correlation with any of the photosynthetic physiological or population growth indicators.

#### 3.6.2 Analysis of equation fitting for the physiological growth characteristics of winter wheat

**Figure 12:**
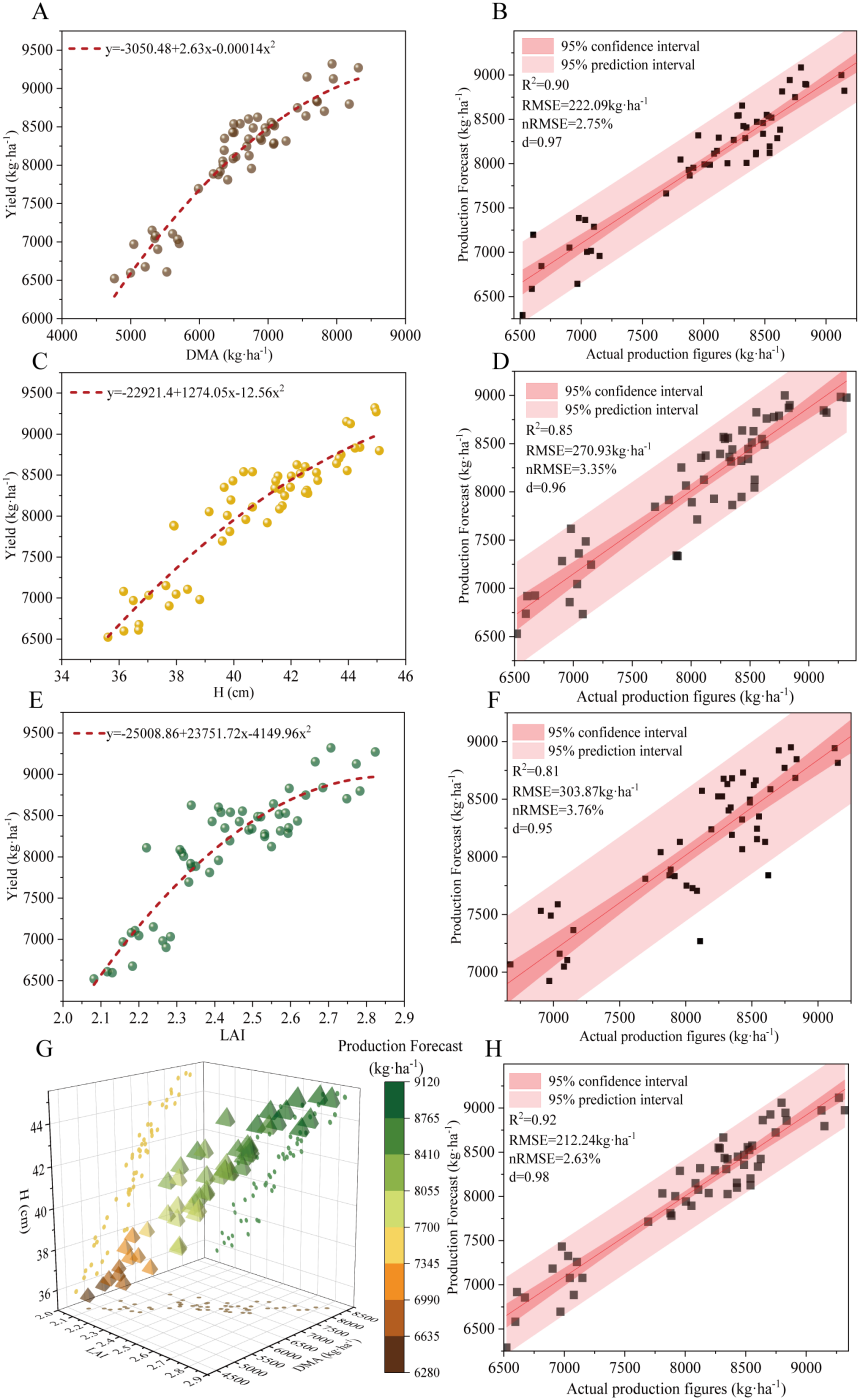
Analysis of the fitting of physiological growth models for winter wheat. **(A, B)** show the fitting and validation of the dry matter accumulation model for winter wheat. **(C, D)** show the fitting and validation of the plant height model for winter wheat. **(E, F)** show the fitting and validation of the leaf area index model for winter wheat. **(G, H)** show the fitting and validation of the multivariate stepwise regression model for winter wheat.

Based on the results of prior PCA dimensionality reduction and correlation analysis, three physiological growth indicators highly correlated with yield—leaf area index (LAI), dry matter accumulation (DMA), and plant height (H)—were selected for fitting and analyzing the yield prediction equations. The results of univariate equation fitting indicated that all three indicators exhibited a significant quadratic relationship with yield (**Figures 12A–F**). The R² values for DMA, H, and LAI, from highest to lowest, were 0.90, 0.85, and 0.81, respectively. Considering the multicollinearity among the physiological growth indicators within the population, a stepwise regression method was employed to analyze the relationships between yield and DMA, LAI, and H, thereby constructing a multivariate prediction equation (**Figures 12G, H**). The results of the equation accuracy test (**Table 2**) indicate that this multiple stepwise regression equation has a good fitting effect. Compared with single indicators, the R² of the comprehensive model increased to 0.92, while the RMSE and nRMSE decreased to 212.24 kg·ha^-1^ and 2.63%, respectively, and the consistency index d reached as high as 0.98. The scatter plot of observed and simulated yields shows a tight and uniform distribution along the 1:1 reference line and within the 95% confidence interval, with no obvious systematic bias. In summary, the physiological growth characteristic fitting equation constructed using LAI, DMA, and H exhibits high fitting accuracy, providing a reliable mathematical model to support the optimization of WFPSR schemes for winter wheat.

**Table 2:** Fitting equations for physiological growth characteristics of winter wheat.

No	Fitted equations	R2	RMSE (kg·ha-1)	nRMSE (%)	d
1	Y= -3050.48 + 2.63D - 0.00014D²	0.90	222.09	2.75	0.97
2	Y = -22921.4 + 1274.05H - 12.56H²	0.85	270.93	3.35	0.96
3	Y = -25008.86 + 23751.72A - 4149.96A²	0.81	303.87	3.76	0.95
4	Y = -27750.13 + 14326.88A + 756.20H - 342.58(A×H) + 0.0158(D×H)	0.92	212.24	2.63	0.98

Y represents the estimated yield; D represents the matter accumulation; H represents the plant height; A represents the leaf area index.

#### 3.6.3 Analysis of winter wheat yield and its components

The primary components of wheat yield include the number of spikes, grains per spike, and thousand-grain weight. The real-time irrigation level (W) and the fertilizer-pesticide treatment regimen (R), as well as their interaction (W×R), had significant effects (P< 0.05) on thousand-grain weight, grains per spike, and final yield, but did not significantly affect the effective panicle number (P > 0.05) (**Table 3**). Under the same fertilizer-pesticide treatment, the winter wheat yield and its component indicators in the W2 real-time irrigation treatment were generally lower than those in the W1 real-time irrigation treatment. During the 2023-2025, the TGW, GNPP, and yield of the W1 treatment increased by 8.58%, 2.96%, and 10.78%, respectively, compared to the W2 treatment. Under constant real-time irrigation levels, as the pesticide reduction ratio increased in the fertilizer-pesticide treatment, the thousand-grain weight and total yield initially remained at high levels before declining simultaneously. Yield and key yield components generally followed this order across the two growing seasons: R4< R3< R1< R2. In 2023-2024, the R2 treatment showed increases of 13.13%, 1.13%, and 14.20% in TGW, EPN, and yield, respectively, compared to the R4 treatment; in 2024-2025, these values increased significantly by 9.88%, 7.09%, and 14.73%, respectively. Over these two years, compared with the CK treatment, the W1R2 treatment showed increases of 6.71%, 2.51%, and 8.23% in 1,000-grain weight, grains per spike, and yield, respectively, while there was no significant difference in the number of effective spikes. In summary, the WFPSR model (W1R2), which combines an 80%θ*_fc_*-60%θ*_fc_* real-time irrigation regime with moderate pesticide reduction and microbial biofertilizer application, maintains a certain yield advantage over CK while achieving water and fertilizer savings of 40.2% and pesticide savings of 30%, respectively.

**Table 3:** Winter wheat yield and its components.

Year	Treatment	Thousand-grain weight (g)	Grain number per panicle	Effective panicle number (×10000·ha^-1^)	Yield (kg·ha-1)	Water and Fertilizer Conservation Rate (%)	Reduction in Pesticide Use (%)
2023-2024	W1R1	41.5a	35.9a	575.5a	8574.09ab	40.34	27
	W1R2	41.8a	36.1a	573.1a	8647.96a	40.34	36
	W1R3	39.2cd	35.9a	583.5a	8211.48ab	40.34	45
	W1R4	38.9c	36.2a	579.2a	8156.18ab	40.34	54
	W2R1	40.2bc	35.8ab	581.4a	8367.27ab	31.19	27
	W2R2	40.9ab	35.6ab	578.8a	8427.56ab	31.19	36
	W2R3	35.1e	34.1c	574.2a	6872.66c	31.19	45
	W2R4	34.2e	34.7bc	572.7a	6796.46c	31.19	54
	CK	40.1cd	35.1ab	578.6a	8143.85b	0	0
	W	*	*	ns	*	-	-
	R	*	*	ns	*	-	-
	W×R	*	*	ns	*	-	-
2024-2025	W1R1	42.3a	36.8a	576.3a	8970.92a	41.07	27
	W1R2	42.5a	37.5a	573.4a	9138.56a	41.07	36
	W1R3	40.1b	35.7bc	582.7a	8341.75b	41.07	45
	W1R4	39.8bc	34.9c	576.1a	8002.14b	41.07	54
	W2R1	39.7bc	35.4bc	585.6a	8229.91b	30.96	27
	W2R2	39.8bc	36.5ab	578.5a	8403.87b	30.96	36
	W2R3	35.3d	34.4c	574.4a	7035.88c	30.96	45
	W2R4	35.1d	34.2c	575.2a	7288.42c	30.96	54
	CK	38.9c	36.7ab	580.7a	8290.25b	0	0
	W	*	*	ns	*	-	-
	R	*	*	ns	*	-	-
	W×R	*	*	ns	*	-	-

Different lowercase letters following data in the same column indicate statistically significant differences at the 0.05 level. W, real-time irrigation level; R, fertilizer-pesticide treatment; *p<0.05, **p<0.01. “-” indicates no association.

## 4 Discussion

### 4.1 Winter wheat physiological growth characteristics

Photosynthesis is the core driver of crop metabolism and energy conversion; its efficiency directly determines the potential for biomass accumulation and the formation of final yield (Dechant et al., 2020; Ding et al., 2024). This study found that under WFPSR, the low-irrigation real-time irrigation treatment had a promoting effect on the photosynthetic physiological indicators of winter wheat. Under the same fertilizer-pesticide treatment, the Pn, SPAD and Gs were generally higher than those in the high-irrigation treatments, exceeding the W2 treatment by 19.75%, 9.93%, and 21.22%, respectively. This is consistent with the findings of Guerfel et al. (2009), who reported that moderate water stress can induce plants to reduce water loss from leaves while maintaining or even enhancing photosynthetic carbon fixation capacity and chlorophyll density per unit leaf area. Studies by Ru et al. (2025) found that moderate water deficit acts as a positive stress signal, improving leaf photosynthetic parameters and pigment accumulation while stimulating root vitality and the antioxidant enzyme system, thereby delaying leaf senescence. To some extent, this also explains why the W1 treatment in this study was able to maintain superior photosynthetic efficiency throughout the growing season while reducing irrigation volume. Unlike most previous studies that have focused solely on the effects of a single stressor on crop photosynthesis (Kamran et al., 2018; Sharma et al., 2025), this study found that the photosynthetic physiological performance of the moderate pesticide reduction combined with microbial fertilizer (R2 treatment) was significantly superior to that of the conventional pesticide application (CK). Some researchers have found that traditional high-dose chemical pesticides induce a massive accumulation of reactive oxygen species within leaves and trigger lipid peroxidation in chloroplast thylakoid membranes, leading to passive stomatal closure and photosynthetic inhibition due to accelerated chlorophyll degradation (Petit et al., 2012). At the same time, existing research indicates that the application of microbial biofertilizers not only helps improve the rhizosphere microecology but also promotes crop photosynthesis and growth, while delaying leaf senescence in the later stages of the growing season (Sharma et al., 2019; Kumar et al., 2022). In the study, the R2 treatment showed significant increases in Pn and Gs of 24.88% and 16.00%, respectively, compared to the CK treatment, and the SPAD values across the two growing seasons were also 10.07% higher than those of the CK treatment, consistent with the results of previous studies. The physiological advantages observed in the R2 treatment may be attributed to the combined regulatory effects of reduced pesticide use, which minimizes chemical damage, and the application of microbial fertilizer, which enhances physiological resistance. This study also found that excessive reduction in pesticide use is equally detrimental to plant growth. When microbial biofertilizers were applied, a significant decline in growth indices was observed when pesticide use was reduced by more than 33%. During critical growth stages in the R4 treatment, H and DMA were reduced by 10.95% and 19.82%, respectively, compared to the R2 treatment across the two growing seasons, while LAI decreased by 8.74% and 14.40% in 2023–2024 and 2024–2025, respectively. Dias (2012) indicated that although pesticides may limit carbon assimilation and inhibit crop growth due to their potential phytotoxicity, their protective role in effectively controlling diseases and preventing crops from suffering greater biotic stress remains irreplaceable. Since the R2–R4 treatments combined the application of microbial biofertilizers with reduced chemical pesticide use, the photosynthetic and growth advantages exhibited by the R2 treatment compared to R3 and R4 cannot be attributed solely to reduced disease pressure. This positive physiological and growth response may result from a combination of factors: reduced biostress due to moderate pesticide application, improvements in the rhizosphere microecology and growth-promoting effects of microbial biofertilizers, or synergistic interactions between these two factors. However, this mechanism requires further validation through future studies incorporating specific plant protection data, such as disease incidence rates and disease severity indices.

### 4.2 Development of the Logistic growth model for winter wheat

The development of crop growth models can further provide effective support for the quantitative analysis of population dynamics and the implementation of precision management (Wang et al., 2018). Previous studies have shown that methods based on the Logistic model to couple crop growth with yield relationships have been applied and validated in various crops, including wheat, maize, and tomato (Baar et al., 2024), demonstrating high model reliability. In this study, using accumulated temperature as the independent variable, the Logistic and Modified Logistic models were employed to simulate the growth processes of winter wheat leaf area index, plant height, and dry matter accumulation under WFPSR. The fitting results for each treatment were satisfactory, effectively describing the growth dynamics of winter wheat. By calculating the derivatives of the Logistic model, numerous biologically meaningful characteristic parameters can be obtained, enabling quantitative analysis of changes in crop growth dynamics. This study found that the treatment involving moderate pesticide reduction combined with microbial biofertilizer application (R2) significantly optimized the wheat growth process, shortening the duration of the rapid growth phase for plant height and dry matter accumulation by 23.91 °C·d and 35.08 °C·d, respectively, over a two-year period compared to the treatment with excessive pesticide reduction (R4). Under the W1 irrigation level, the maximum leaf area expansion rate and maximum plant height growth rate were 13.61% and 7.11% higher, respectively, than those of the W2 treatment. Compared with the CK treatment, the maximum dry matter accumulation rate and maximum leaf area expansion rate under W1R2 increased by 14.09% and 25.27%, respectively. This may be related to the combined effects of reduced pesticide toxicity, the growth-promoting action of microbial fertilizers, and the stress response induced by moderate moisture levels, enabling plants to achieve higher biomass production efficiency within a shorter accumulated temperature period. Compared to previous Logistic models that focused solely on water and fertilizer responses (Archontoulis and Miguez, 2015), this study extends the model to include WFPSR and verifies its applicability in complex cropping systems. However, the current model relies primarily on macroclimatic parameters such as accumulated temperature, whereas actual crop growth in the field is influenced by the combined effects of various interacting factors, including soil fertility and cultivation practices. Future research may consider incorporating relative normalization methods into the model to eliminate biases caused by differences in total accumulated temperature, thereby deriving more universal patterns of crop growth and biomass accumulation and enhancing the model’s applicability across different ecological regions and cultivation management practices.

### 4.3 Quantitative analysis of the relationship between winter wheat yield and physiological growth

In this study, winter wheat yield varied significantly with the optimization of real-time water-fertilizer-pesticide regulation; specifically, yield increased significantly under the treatment combining moderate real-time irrigation with a 33% reduction in pesticide use and microbial biofertilizer application (W1R2). Compared to the local conventional management (CK) and the high-intensity real-time irrigation treatment (W2R2), the yield of W1R2 increased by 8.23% and 5.67%, respectively, and was significantly superior to the treatment with a high proportion of pesticide reduction. This is consistent with the findings of studies by Liu et al. (2024) regarding the effects of different irrigation strategies on winter wheat yield. Moderate water deficit during the winter wheat growing season has minimal negative impact on final yield; conversely, sustained high irrigation rates can lead to root hypoxia, exerting a certain inhibitory effect on crop physiological growth (Ploschuk et al., 2018). This study found that real-time coordinated regulation of water, fertilizer, and pesticides significantly affects the 1,000-kernel weight and grains per spike of winter wheat. As key yield components, these two indicators determine total yield. With the application of moderate pesticide reduction and microbial biofertilizer (R2), the TGW and grains per spike increased by 6.71% and 2.51%, respectively, compared to CK. Treatments R3 and R4, which involved excessive pesticide reduction, saw 1,000-grain weight and grains per spike decrease by 5.25% and 6.33%, respectively, compared to CK. Correlation analysis indicated that 1,000-grain weight and grains per spike showed a high, significant positive correlation with physiological growth indicators such as SPAD and LAI. The correlation coefficients between TGW and DMA, LAI, and SPAD were 0.91, 0.80, and 0.69, respectively. Regarding the significant decline in yield resulting from excessive reduction in pesticide application, based on crop physiological growth patterns and previous research, this may be related to increased disease pressure in the field. Research by Wildermuth et al. (2001) indicates that sustained soil moisture and high humidity in the canopy promote the rapid spread and severity of stem base rot. Disease infection disrupts the plant’s vascular system, severely impeding the transport of water and photosynthetic products (Yadeta and Thomma, 2013), thereby affecting crop physiological growth indicators. This is consistent with the impaired physiological growth observed in the W2R3 and W2R4 treatments in this study. In contrast, the W1R2 treatment achieved higher yields while maintaining lower pesticide application rates. This may be due not only to the fact that the application of moderate amounts of chemical pesticides combined with microbial biofertilizers mitigated the chemical damage induced by high pesticide doses while maintaining effective disease control, but also to the fact that its appropriate real-time irrigation level (W1) stimulated the plants’ own stress resistance and may have suppressed the spread of potential diseases by reducing field humidity. This synergistic interaction among water, fertilizer, and pesticides may be the key to maintaining high photosynthetic physiological activity in the leaves. Meanwhile, the formation of wheat thousand-kernel weight is highly dependent on the photosynthetic physiological activity maintained in the canopy after flowering; maintaining indicators such as SPAD and LAI can effectively delay flag leaf senescence, significantly promote the transport and redistribution of dry matter to the grains, extend the active grain-filling period to increase grain weight, and boost yield (Li et al., 2025). In summary, winter wheat yield and its components are influenced by the combined effects of different irrigation levels, pesticide application rates, and the use of microbial biofertilizers. Since this study lacks supporting phytosanitary data, such as disease severity indices, and because the reduction in pesticide use and the application of microbial biofertilizers were coupled in the experimental design, future research should combine field disease monitoring with an orthogonal experimental design to clarify the independent contributions of each individual factor and their respective disease control effects.

## 5 Conclusion

Compared with traditional empirical water, fertilizer, and pesticide management, the appropriate WFPSR treatment (W1R2) significantly improved the photosynthetic physiology and growth traits of winter wheat. An improved logistic model based on accumulated temperature accurately captured the crop’s dynamic developmental process, confirming that the W1R2 synergistic regulation model enables winter wheat to reach peak dry matter accumulation and leaf area expansion within a shorter period of effective accumulated temperature. Analysis using PCA dimensionality reduction and correlation analysis confirmed that this model primarily increases yield by improving key physiological growth indicators such as SPAD and DMA, thereby increasing thousand-kernel weight and grains per spike. The combined water and fertilizer savings rate and the pesticide savings rate were 40.2% and 33%, respectively, compared to CK, and the final yield was 8.23% higher than that of CK.

This study provides a relatively precise field management framework for the green and efficient production of winter wheat in the North China Plain and similar ecological regions, offering valuable insights for promoting the transition of agricultural systems toward sustainable models and mitigating non-point source pollution in farmland. Given that this experiment was conducted in a relatively fixed study area, future research could validate these findings through multi-year, multi-site field trials conducted across broader ecological and climatic regions.

## Data Availability

The original contributions presented in the study are included in the article/**Supplementary Material**. Further inquiries can be directed to the corresponding author.
