# Supplementary material for: Study on changes in the physiological growth characteristics and yield-increasing effects of winter wheat under water-fertilizer-pesticide synergistic regulation
Source: Front Plant Sci. 2026 Jul 1;17:1865682. doi: 10.3389/fpls.2026.1865682 (PMC13368560; doi:10.3389/fpls.2026.1865682)
Supplement: Supplementary file 1 [file SupplementaryFile1.docx]

Supplementary Material

（**Data support for the Figures in the chapters**）

# 3 Results

## 3.1 Relative chlorophyll values in winter wheat leaves

**Figure 3** Relative chlorophyll content in winter wheat leaves at various growth stages during the 2023-2024 and 2024-2025 growing seasons

| Year | Growth stage | SPAD | | | | | | | | |
| --- | --- | --- | --- | --- | --- | --- | --- | --- | --- | --- |
|  |  | W1R1 | W1R2 | W1R3 | W1R4 | W2R1 | W2R2 | W2R3 | W2R4 | CK |
| 2023-2024 | ES | 33.97 | 38.47 | 38.37 | 38.7 | 30.73 | 35.57 | 35.43 | 35.43 | 35.80 |
|  | TS | 42.3 | 45.37 | 44.57 | 45.33 | 36.5 | 40.53 | 39.97 | 39.9 | 36.15 |
|  | RS | 44.87 | 48.33 | 47.73 | 48.17 | 42.8 | 46.43 | 46.03 | 44.03 | 44.33 |
|  | JS | 49.4 | 52.53 | 52.07 | 51.4 | 47.83 | 50.87 | 47.8 | 47.3 | 49.40 |
|  | GFS | 53.37 | 55.67 | 49.93 | 46.73 | 50.27 | 51.4 | 42.73 | 39.57 | 48.77 |
|  | MS | 10.3 | 12.27 | 8.43 | 6.53 | 8.57 | 10.3 | 4.77 | 3.23 | 7.10 |
| 2024-2025 | ES | 36.5 | 39.47 | 39.13 | 39.3 | 33.3 | 37.83 | 38.13 | 37.83 | 32.53 |
|  | TS | 43.17 | 46.47 | 46.5 | 46.13 | 38.07 | 41.93 | 41.2 | 42.07 | 38.70 |
|  | RS | 46.53 | 50.1 | 49.57 | 49.17 | 43.97 | 47.57 | 47.57 | 46.8 | 45.07 |
|  | JS | 51.33 | 54.2 | 53.53 | 52.63 | 48.43 | 51.4 | 44.6 | 46.3 | 50.67 |
|  | GFS | 54.57 | 57.4 | 50.67 | 47.73 | 52.1 | 53.87 | 41.73 | 37.73 | 52.20 |
|  | MS | 9.5 | 9.4 | 7.67 | 5.43 | 8.03 | 9.8 | 4 | 2.4 | 7.73 |

## 3.3 Leaf area index of winter wheat

## Figure 5 Dry matter accumulation in winter wheat at various growth stages during the 2023-2024 and 2024-2025 growing seasons

| Year | Growth stage | LAI | | | | | | | | |
| --- | --- | --- | --- | --- | --- | --- | --- | --- | --- | --- |
|  |  | W1R1 | W1R2 | W1R3 | W1R4 | W2R1 | W2R2 | W2R3 | W2R4 | CK |
| 2023-2024 | ES | 0.37 | 0.29 | 0.24 | 0.28 | 0.28 | 0.24 | 0.27 | 0.22 | 0.21 |
|  | TS | 0.65 | 0.78 | 0.75 | 0.62 | 0.64 | 0.71 | 0.69 | 0.67 | 0.47 |
|  | RS | 3.24 | 3.35 | 2.35 | 2.92 | 2.81 | 2.86 | 2.54 | 2.43 | 3.32 |
|  | JS | 5.19 | 5.25 | 4.92 | 4.83 | 4.97 | 4.99 | 4.31 | 4.36 | 4.87 |
|  | GFS | 4.35 | 4.43 | 4.16 | 3.99 | 4.05 | 3.98 | 3.62 | 3.64 | 4.05 |
|  | MS | 2.29 | 2.35 | 2.28 | 2.2 | 2.2 | 2.14 | 1.99 | 1.94 | 2.18 |
| 2024-2025 | ES | 0.31 | 0.30 | 0.28 | 0.21 | 0.24 | 0.27 | 0.22 | 0.24 | 0.22 |
|  | TS | 0.64 | 0.67 | 0.76 | 0.72 | 0.68 | 0.73 | 0.67 | 0.76 | 0.57 |
|  | RS | 3.21 | 3.32 | 2.33 | 2.89 | 2.78 | 2.39 | 2.38 | 2.27 | 3.29 |
|  | JS | 5.14 | 5.2 | 4.87 | 4.78 | 4.92 | 4.94 | 4.3 | 4.32 | 4.82 |
|  | GFS | 4.31 | 4.37 | 4.21 | 3.98 | 4.01 | 3.94 | 3.77 | 3.73 | 4.01 |
|  | MS | 2.27 | 2.33 | 2.26 | 2.24 | 2.18 | 2.12 | 1.97 | 1.92 | 2.16 |

## 3.3.2 Temporal dynamics in winter wheat leaf area index based on the improved Logistics model

**Figure 6** Fitting parameters and characteristic parameters of the improved logistic model for leaf area index of winter wheat in 2023–2024 and 2024–2025

| Year | Treatment | Fitting Parameters | | | | R² | Characteristic Parameters | | |
| --- | --- | --- | --- | --- | --- | --- | --- | --- | --- |
|  |  | c | d | f | g |  | C_xinf_ | C_Rmax_ | C_xmax_ |
| 2023-2024 | W1R1 | 7.54 | 0.0000018 | -0.0063 | 4.93 | 0.99 | 878.86 | 0.0048 | 1698.37 |
|  | W1R2 | 7.64 | 0.0000020 | -0.0068 | 5.05 | 0.99 | 853.27 | 0.0056 | 1698.49 |
|  | W1R3 | 8.99 | 0.0000020 | -0.0064 | 5.62 | 0.97 | 954.54 | 0.0040 | 1618.69 |
|  | W1R4 | 7.78 | 0.0000019 | -0.0064 | 5.32 | 0.99 | 938.47 | 0.0045 | 1711.23 |
|  | W2R1 | 7.56 | 0.0000020 | -0.0068 | 5.08 | 0.97 | 858.41 | 0.0056 | 1709.60 |
|  | W2R2 | 7.07 | 0.0000019 | -0.0069 | 5.14 | 0.95 | 866.93 | 0.0056 | 1783.85 |
|  | W2R3 | 7.63 | 0.0000019 | -0.0065 | 5.24 | 0.97 | 918.56 | 0.0048 | 1720.74 |
|  | W2R4 | 8.47 | 0.0000019 | -0.0062 | 5.22 | 0.96 | 925.31 | 0.0046 | 1677.42 |
|  | CK | 8.73 | 0.0000019 | -0.0063 | 5.39 | 0.99 | 952.65 | 0.0046 | 1693.55 |
| 2024-2025 | W1R1 | 6.17 | 0.0000019 | -0.0068 | 4.58 | 0.99 | 763.53 | 0.0056 | 1772.25 |
|  | W1R2 | 5.97 | 0.0000020 | -0.0072 | 4.73 | 0.98 | 748.57 | 0.0058 | 1770.94 |
|  | W1R3 | 7.76 | 0.0000016 | -0.0058 | 4.75 | 0.98 | 913.54 | 0.0048 | 1822.78 |
|  | W1R4 | 5.37 | 0.0000021 | -0.0076 | 5.06 | 0.99 | 774.77 | 0.0055 | 1793.84 |
|  | W2R1 | 6.10 | 0.0000019 | -0.0066 | 4.71 | 0.97 | 806.48 | 0.0051 | 1786.49 |
|  | W2R2 | 5.66 | 0.0000021 | -0.0074 | 5.07 | 0.97 | 795.27 | 0.0054 | 1782.61 |
|  | W2R3 | 5.73 | 0.0000017 | -0.0062 | 4.54 | 0.99 | 813.8 | 0.0044 | 1793.10 |
|  | W2R4 | 6.55 | 0.0000016 | -0.0056 | 4.40 | 0.97 | 847.41 | 0.0042 | 1781.65 |
|  | CK | 6.03 | 0.0000019 | -0.0066 | 4.88 | 0.98 | 839.47 | 0.0045 | 1710.94 |

## 3.4 Plant height characteristics in winter wheat

**Figure 7** Winter wheat plant height at various growth stages in 2023-2024 and 2024-2025

| Year | Growth stage | H（cm） | | | | | | | | |
| --- | --- | --- | --- | --- | --- | --- | --- | --- | --- | --- |
|  |  | W1R1 | W1R2 | W1R3 | W1R4 | W2R1 | W2R2 | W2R3 | W2R4 | CK |
| 2023-2024 | ES | 0.37 | 0.29 | 0.24 | 0.28 | 0.28 | 0.24 | 0.27 | 0.22 | 0.21 |
|  | TS | 0.65 | 0.78 | 0.75 | 0.62 | 0.64 | 0.71 | 0.69 | 0.67 | 0.47 |
|  | RS | 3.24 | 3.35 | 2.35 | 2.92 | 2.81 | 2.86 | 2.54 | 2.43 | 3.32 |
|  | JS | 5.19 | 5.25 | 4.92 | 4.83 | 4.97 | 4.99 | 4.31 | 4.36 | 4.87 |
|  | GFS | 4.35 | 4.43 | 4.16 | 3.99 | 4.05 | 3.98 | 3.62 | 3.64 | 4.05 |
|  | MS | 2.29 | 2.35 | 2.28 | 2.2 | 2.2 | 2.14 | 1.99 | 1.94 | 2.18 |
| 2024-2025 | ES | 0.31 | 0.30 | 0.28 | 0.21 | 0.24 | 0.27 | 0.22 | 0.24 | 0.22 |
|  | TS | 0.64 | 0.67 | 0.76 | 0.72 | 0.68 | 0.73 | 0.67 | 0.76 | 0.57 |
|  | RS | 3.21 | 3.32 | 2.33 | 2.89 | 2.78 | 2.39 | 2.38 | 2.27 | 3.29 |
|  | JS | 5.14 | 5.2 | 4.87 | 4.78 | 4.92 | 4.94 | 4.3 | 4.32 | 4.82 |
|  | GFS | 4.31 | 4.37 | 4.21 | 3.98 | 4.01 | 3.94 | 3.77 | 3.73 | 4.01 |
|  | MS | 2.27 | 2.33 | 2.26 | 2.24 | 2.18 | 2.12 | 1.97 | 1.92 | 2.16 |

## 3.4.2 Temporal dynamics in winter wheat plant height based on the Logistics mode

**Figure 8**. Fitting parameters and characteristic parameters of the logistic model for dry matter accumulation in winter wheat during 2023-2025

| Year | Treatment | Model Parameters | | | R^2^ | Feature Parameters | | | |
| --- | --- | --- | --- | --- | --- | --- | --- | --- | --- |
|  |  | L | a | b |  | V_1_ | GDD_1_ | GDD_2_ | GDD_3_ |
| 2023-2024 | W1R1 | 73.09 | 0.003 | 2.84 | 0.99 | 0.055 | 938.89 | 502.81 | 1374.97 |
|  | W1R2 | 73.15 | 0.0032 | 2.88 | 0.99 | 0.058 | 911.6 | 494.84 | 1328.36 |
|  | W1R3 | 68.07 | 0.0031 | 2.95 | 0.98 | 0.052 | 956.88 | 529.3 | 1384.46 |
|  | W1R4 | 67.3 | 0.003 | 2.8 | 0.98 | 0.05 | 949.41 | 502.98 | 1395.84 |
|  | W2R1 | 68.36 | 0.0031 | 2.86 | 0.98 | 0.053 | 922.27 | 497.44 | 1347.1 |
|  | W2R2 | 70.06 | 0.0032 | 2.96 | 0.98 | 0.056 | 925.76 | 514.21 | 1317.31 |
|  | W2R3 | 63.17 | 0.003 | 2.8 | 0.97 | 0.047 | 934.9 | 494.45 | 1375.35 |
|  | W2R4 | 62.42 | 0.0029 | 2.8 | 0.98 | 0.045 | 968.46 | 512.77 | 1324.15 |
|  | CK | 67.9 | 0.0033 | 3.01 | 0.97 | 0.056 | 906.33 | 509.66 | 1393 |
| 2024-2025 | W1R1 | 74.6 | 0.003 | 3.05 | 0.99 | 0.056 | 1015.46 | 576.47 | 1454.45 |
|  | W1R2 | 75.22 | 0.0033 | 3.34 | 0.97 | 0.062 | 1006.88 | 610.21 | 1403.55 |
|  | W1R3 | 69.27 | 0.0031 | 3.2 | 0.98 | 0.053 | 1039.81 | 612.23 | 1467.39 |
|  | W1R4 | 69.07 | 0.003 | 3.05 | 0.98 | 0.051 | 1026.57 | 583.15 | 1469.99 |
|  | W2R1 | 69.88 | 0.003 | 3.06 | 0.99 | 0.053 | 1007.91 | 574.7 | 1441.12 |
|  | W2R2 | 70.85 | 0.0033 | 3.2 | 0.98 | 0.058 | 980.81 | 576.84 | 1384.78 |
|  | W2R3 | 62.52 | 0.0031 | 3.04 | 0.97 | 0.048 | 983.74 | 557.54 | 1409.94 |
|  | W2R4 | 62.24 | 0.0031 | 3.12 | 0.97 | 0.048 | 1019 | 588.62 | 1449.38 |
|  | CK | 69.46 | 0.0033 | 3.19 | 0.97 | 0.057 | 974.82 | 572.08 | 1377.56 |

## 3.5 Dry matter accumulation in winter wheat

**Figure 9** Dry matter accumulation in winter wheat at various growth stages during the 2023-2024 and 2024-2025 growing seasons

| Year | Growth stage | DMA（kg/ha） | | | | | | | |
| --- | --- | --- | --- | --- | --- | --- | --- | --- | --- |
|  |  | W1R1 | W1R2 | W1R3 | W1R4 | W2R1 | W2R2 | W2R3 | W2R4 |
| 2023-2024 | ES | 87.87 | 85.85 | 89.89 | 85.85 | 85.85 | 88.98 | 85.85 | 89.81 |
|  | TS | 1026.86 | 1016.66 | 919.11 | 1099.91 | 971.62 | 958.49 | 836.28 | 1057.47 |
|  | RS | 2889.62 | 3237.05 | 2408.87 | 2272.51 | 2774.47 | 2488.64 | 1995.76 | 1910.92 |
|  | JS | 7307.35 | 7943.65 | 5451.98 | 5154.03 | 6977.08 | 6910.42 | 5653.98 | 5264.12 |
|  | GFS | 14287.49 | 14374.32 | 12171.51 | 11613.99 | 11922.07 | 12427.71 | 10181.75 | 10212.21 |
|  | MS | 20462.63 | 21212.1 | 18568.88 | 18117.81 | 16612.59 | 17671.64 | 13515.08 | 14617.14 |
| 2024-2025 | ES | 69 | 72.11 | 77.31 | 72.97 | 71.26 | 84.13 | 74.69 | 68.72 |
|  | TS | 1059.33 | 1032.9 | 977.66 | 958.19 | 1028.71 | 1049 | 968.31 | 1013.94 |
|  | RS | 2795.25 | 3205.21 | 2287.18 | 2684.1 | 2747.22 | 2464.08 | 1976.26 | 1892.19 |
|  | JS | 7235.21 | 7865.19 | 5398.27 | 5103.17 | 6908.07 | 6842.23 | 5598.07 | 5212.11 |
|  | GFS | 13816.21 | 14232.19 | 12051.29 | 11499.04 | 11474.21 | 12304.93 | 9807.28 | 9121.32 |
|  | MS | 19663.65 | 19877.24 | 18421.87 | 18329.95 | 17817.37 | 18097.08 | 13674.78 | 13080.82 |

## 3.5.2 Temporal dynamics of dry matter accumulation in winter wheat based on the Logistics model

**Figure 10**. Fitting parameters and characteristic parameters of the Logistic model for dry matter accumulation in winter wheat

| Year | Treatment | Model Parameters | | | R^2^ | Feature Parameters | | | |
| --- | --- | --- | --- | --- | --- | --- | --- | --- | --- |
|  |  | L | a | b |  | V_1_ | GDD_1_ | GDD_2_ | GDD_3_ |
| 2023-2024 | W1R1 | 22073.31 | 0.0021 | 3.84 | 0.99 | 11.588 | 1826.85 | 1199.73 | 2453.97 |
|  | W1R2 | 21934.59 | 0.0021 | 3.73 | 0.99 | 11.57 | 1768.14 | 1143.99 | 2392.29 |
|  | W1R3 | 22011.69 | 0.002 | 4.08 | 0.98 | 11.226 | 2001.45 | 1355.88 | 2647.02 |
|  | W1R4 | 22770.65 | 0.002 | 4.11 | 0.99 | 11.215 | 2084.11 | 1415.6 | 2752.62 |
|  | W2R1 | 21484.61 | 0.0018 | 3.57 | 0.99 | 9.775 | 1962.94 | 1239.34 | 2686.54 |
|  | W2R2 | 20615.09 | 0.002 | 3.78 | 0.99 | 10.462 | 1862.76 | 1214.01 | 2511.51 |
|  | W2R3 | 14945.73 | 0.0022 | 3.76 | 0.99 | 8.033 | 1749.25 | 1136.71 | 2361.79 |
|  | W2R4 | 14803.67 | 0.002 | 3.64 | 0.99 | 7.402 | 1819.46 | 1160.98 | 2477.94 |
|  | CK | 20451.15 | 0.002 | 3.76 | 0.99 | 10.277 | 1868.21 | 1213.01 | 2523.41 |
| 2024-2025 | W1R1 | 23302.02 | 0.0025 | 4.43 | 0.99 | 14.389 | 1792.58 | 1259.4 | 2325.76 |
|  | W1R2 | 24875.24 | 0.0023 | 4.17 | 0.99 | 14.179 | 1828.23 | 1250.62 | 2405.84 |
|  | W1R3 | 22280.68 | 0.0024 | 4.62 | 0.99 | 13.424 | 1915.59 | 1369.13 | 2462.05 |
|  | W1R4 | 22965.72 | 0.0022 | 4.39 | 0.99 | 12.689 | 1986.92 | 1391.01 | 2582.83 |
|  | W2R1 | 18600.57 | 0.0024 | 4.06 | 0.99 | 11.021 | 1714.09 | 1158.41 | 2269.77 |
|  | W2R2 | 19976.82 | 0.0024 | 4.26 | 0.99 | 12.086 | 1760.31 | 1216.11 | 2304.51 |
|  | W2R3 | 14657.33 | 0.0026 | 4.35 | 0.99 | 9.601 | 1659.5 | 1156.84 | 2162.16 |
|  | W2R4 | 16746.58 | 0.0024 | 4.37 | 0.99 | 10.174 | 1797.85 | 1255.89 | 2339.81 |
|  | CK | 20659.7 | 0.0024 | 4.25 | 0.99 | 12.293 | 1785.67 | 1232.33 | 2339.01 |
